# Insights into the Musa genome: Syntenic relationships to rice and between Musa species

**DOI:** 10.1186/1471-2164-9-58

**Published:** 2008-01-30

**Authors:** Magali Lescot, Pietro Piffanelli, Ana Y Ciampi, Manuel Ruiz, Guillaume Blanc, Jim Leebens-Mack, Felipe R da Silva, Candice MR Santos, Angélique D'Hont, Olivier Garsmeur, Alberto D Vilarinhos, Hiroyuki Kanamori, Takashi Matsumoto, Catherine M Ronning, Foo Cheung, Brian J Haas, Ryan Althoff, Tammy Arbogast, Erin Hine, Georgios J Pappas, Takuji Sasaki, Manoel T Souza, Robert NG Miller, Jean-Christophe Glaszmann, Christopher D Town

**Affiliations:** 1 French Agricultural Research Center for International Development (CIRAD), UMR 1096, Avenue Agropolis, TA40/03, FR-34398, Montpellier, Cedex 5, France; 2 The J. Craig Venter Institute, 9704 Medical Center Drive, Rockville MD 20850, USA; 3 Embrapa Genetic Resources and Biotechnology (CENARGEN), P B I., Final Av. W/5 Norte, Asa Norte 70770-900, Caixa-Postal: 02372, Brasília, Brazil; 4 Genomic Sciences and Biotechnology program, Catholic University of Brasília (UCB), SGAN 916, Modulo B, Asa Norte 70790-160, Brasília DF, Brazil; 5 Structural and Genomic Information Laboratory (IGS), C.N.R.S. UPR 2589, Institute of Structural Biology and Microbiology (IBSM), Parc Scientifique de Luminy, 163 avenue de Luminy, FR-13288 Marseille Cedex 9, France; 6 Department of Plant Biology, University of Georgia, Athens, GA 30602, USA; 7 Brazilian Agricultural Research Corporation (EMBRAPA), National Research Center of Cassava and Fruit Crops (CNPMF), P.O Box 007, Zip Code 44380.000, Cruz das Almas BA, Brazil; 8 Rice Genome Research Program (RGP), National Institute of Agrobiological Sciences (NIAS)/Institute of the Society for Techno-innovation of Agriculture, Forestry and Fisheries, Tsukuba, Ibaraki 305-8602, Japan; 9 Structural and Genomic Information Laboratory (IGS), C.N.R.S. UPR 2589, Institute of Structural Biology and Microbiology (IBSM), Parc Scientifique de Luminy, 163 avenue de Luminy, FR-13288 Marseille Cedex 9, France; 10 Parco Tecnologico Padano, Via Einstein, Lodi 26900, Italy

## Abstract

**Background:**

*Musa *species (Zingiberaceae, Zingiberales) including bananas and plantains are collectively the fourth most important crop in developing countries. Knowledge concerning *Musa *genome structure and the origin of distinct cultivars has greatly increased over the last few years. Until now, however, no large-scale analyses of *Musa *genomic sequence have been conducted. This study compares genomic sequence in two *Musa *species with orthologous regions in the rice genome.

**Results:**

We produced 1.4 Mb of *Musa *sequence from 13 BAC clones, annotated and analyzed them along with 4 previously sequenced BACs. The 443 predicted genes revealed that Zingiberales genes share GC content and distribution characteristics with eudicot and Poaceae genomes. Comparison with rice revealed microsynteny regions that have persisted since the divergence of the Commelinid orders Poales and Zingiberales at least 117 Mya. The previously hypothesized large-scale duplication event in the common ancestor of major cereal lineages within the Poaceae was verified. The divergence time distributions for *Musa*-Zingiber (Zingiberaceae, Zingiberales) orthologs and paralogs provide strong evidence for a large-scale duplication event in the *Musa *lineage after its divergence from the Zingiberaceae approximately 61 Mya. Comparisons of genomic regions from *M. acuminata *and *M. balbisiana *revealed highly conserved genome structure, and indicated that these genomes diverged circa 4.6 Mya.

**Conclusion:**

These results point to the utility of comparative analyses between distantly-related monocot species such as rice and *Musa *for improving our understanding of monocot genome evolution. Sequencing the genome of *M. acuminata *would provide a strong foundation for comparative genomics in the monocots. In addition a genome sequence would aid genomic and genetic analyses of cultivated *Musa *polyploid genotypes in research aimed at localizing and cloning genes controlling important agronomic traits for breeding purposes.

## Background

**Figure 1:**
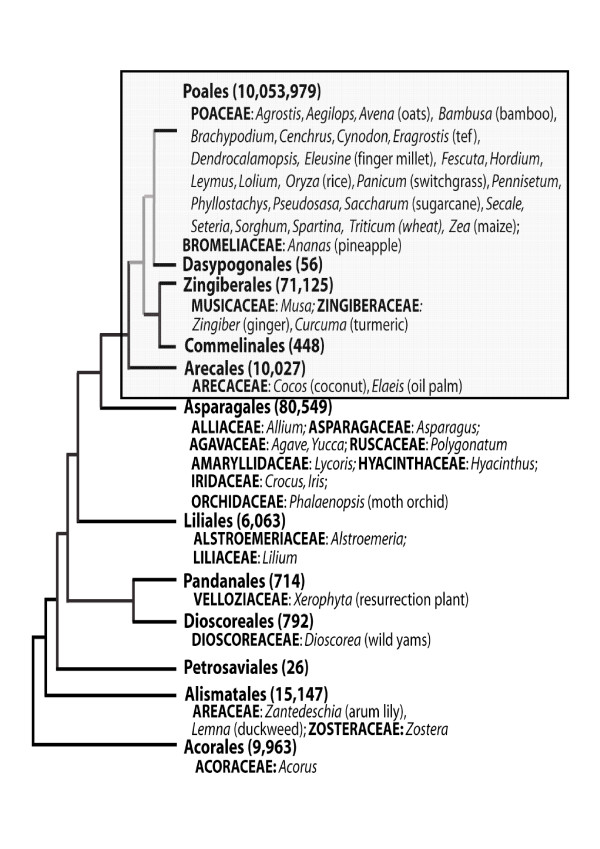
**Current understanding of relationships among monocot orders [118]**. Families are shown in bold caps and genera with EST sequences in dbEST [119]. The number of sequences in GenBank (as of 10/08/07) are shown in parentheses for each order and the shaded box highlights Commelinid orders. The nodes with < 75% bootstrap support are grey.

Taken together, *Musa *species (bananas and plantains) comprise the fourth most important crop in developing countries [1]. The fruit is a staple food in sub-Saharan Africa, South and Central America and much of Asia, while the leaves are used for sheltering and wrapping food and the male bud can be eaten as a vegetable. *Musa *is a member of the monocot order Zingiberales, a Commelinid lineage that diverged from the line leading to rice (Poales) in the mid-cretaceous period over 100 million years ago (Figure 1) [2,3]. The *Musa *species *Musa acuminata *(AA genome) and *Musa balbisiana *(BB genome), both with 2n = 22 chromosomes, represent the two main progenitors of cultivated banana varieties. Table bananas are sterile, parthenocarpic and diploids AA or triploid with the AAA genome constitution, and represent only a fraction of world production, although they are an important cash crop. Cooking bananas and plantain cultivars, mostly consumed in the countries of production, generally have an AAB or ABB genome constitutions [4]; these are boiled, fried, dried, or sometimes ground into flour.

Knowledge concerning the genetic diversity, the origin of cultivars [5-12] and *Musa *genome structure [13-15] has greatly increased over the last few years. The haploid genome of *Musa *species was estimated as varying between 560 to 600 Mb in size [16,17], just four times larger than that of the model plant Arabidopsis (125 Mb) [18] and 30% larger than that of rice (390 Mb) [19]. Genetic maps have been developed [20-23] and recently, BAC resources were generated for both *M. acuminata *[24,25] and *M. balbisiana *[26]. A cytogenetic map based on BAC-FISH is being anchored to genetic maps in order to better characterize structural variation among *M. acuminata *genomes [22]. These resources will pave the way for studies of *Musa *genome structure and evolution through comparisons with other monocot and eudicot genomes.

The utility of genomic comparisons of monocot and eudicot plants (e.g. [27-30]) is growing with the availability of the complete genome sequences of rice [19], Arabidopsis [18] and poplar [31], and active genome sequencing projects for a growing number of other angiosperms [32]. Most genome-scale comparative investigations within the monocots have focused on analyses of closely-related species of monocots belonging to the family of Poaceae [27,33-36]. Numerous papers have described extensive microsynteny between rice, barley, wheat, maize, *Sorghum *and sugarcane [27,35,37-42], although the degree of conservation varies between different chromosomal locations. Fewer attempts have been made to investigate the synteny between distantly-related plants. In addition, whereas extensive genomic resources have been developed for rice and other cereal species in the grass family (Poaceae), there is relatively little data on gene content or genome structure for non-grass monocots (Figure 1). Recently, the first two BAC clones genomic sequences [43], and a BAC end sequencing study of the *M. acuminata *genome [44] have been published. Here we present data on the genomic structure and organization of 1.8 Mb of *Musa *genomic nuclear DNA (including the two BAC sequenced previously [43]), show for the first time the existence of microsynteny between *Musa*, rice and Arabidopsis, characterize the extent of microsynteny between the two *Musa *species representing the progenitors of most cultivated genotypes, analyze monocot EST sequences and discuss the evolutionary implication of these results. The BAC clones sequenced in this study were identified by hybridization with gene sequences previously selected to correspond to one or a few loci in *Musa*, rice and Arabidopsis, thus possibly contain orthologous sequences with these distantly related plant species.

## Results

### Selection of *Musa *BAC clones using broad-spectrum *Sorghum *cDNA and *Musa *RFLP probes

As part of a program aiming at selecting conserved probes from monocotyledons and *Arabidopsis thaliana *towards comparative genetic mapping studies, genomic and cDNA probes from various species were tested by Southern hybridization on DNA of various monocotyledons including *Musa *and rice. Among the probes found conserved between rice, *M. acuminata *cv. Madang, *M. balbisiana *cv. Pisang Klutuk Wulung (PKW) and Arabidopsis that revealed a single or low copy locus hybridization pattern, nine were selected. These nine probes that correspond to *Sorghum bicolor *cDNAs (SbRPG) were used to screen a *M. acuminata *cv. Calcutta-4 bacterial artificial chromosome (BAC) library (Table 1 and Additional file 1). Of these nine SbRPG genes, four encoded nuclear genes targeted to the chloroplast and/or implicated in photosynthetic-related functions, supporting the notion that this class of genes is under strong pressure for functional conservation. All *Musa *BACs identified were subjected to HindIII fingerprinting. This enabled us to separate the *Musa *BACs into groups likely to be derived from different regions of the *Musa *genome. Overall, a good correlation was observed between the number of loci identified in rice, *Sorghum *and *Musa *by Southern blot, BAC fingerprint (for *Musa*) and analysis of whole genome sequence (for rice) for these nine SbRPG genes (Table 1), all of which were found to be in single or low-copy in both *Musa *and rice.

**Table 1:** List of probes used to identify the *Musa *BAC clones sequenced as part of the present study. Estimated copy numbers of these sequences in rice, *Sorghum *and *Musa *are indicated for SbRPG (*Sorghum bicolor*) sequences. MA4 are BAC clones from *M. acuminata *cv. Calcutta-4 and MBP are BAC clones from *M. balbisiana *cv. Pisang Klutuk Wulung.

Probe name and AC number*	**Putative function**	**Estimated copy number in rice by Blast analysis (Rice genes locus identifier)**	**Estimated copy number in *Sorghum *by Southern blot analysis**	**Estimated copy number in *Musa *by Southern blot analysis**	**Number of identified *Musa *BAC clones**	**Number of *Musa *BAC fingerprint groups**	*Musa *BAC clones sequenced (size) and AC number*
**SbRPG132** DQ185891	chlorophyll A-B binding protein type I	**6** Os01g41710.1 Os09g17740.1 Os01g52240.1 Os03g39610.1 Os07g37550.1 Os11g13890.2	4	more than 4	23	6	MA4_25J11 (105019 bp) AC186746
**SbRPG373** DQ185892	hypothetical protein	**1** Os07g02340.1	1	1	29	2	MA4_64C22 (80932 bp) AC186752
							MA4_8L21 (115790 bp) AC186748
**SbRPG661** DQ185893	thioredoxin	**2** Os10g34520.1 Os07g10250.1	2	2–3	20	2	MA4_54B05 (54106 bp) AC186753
							MA4_78I12 (150982 bp) AC186750
**SbRPG748** DQ185894	porphobilinogen deaminase	**1** Os07g10250.1	1	1	1	1	MA4_42M13 (29567 bp) AC186749
**SbRPG851** DQ185895	phosphoglycerate kinase	**2** Os05g41640.1 Os01g58610.1	2	2–3	12	2	MA4_112I10 (102441 bp) AC186756
							MA4_106O17 (143796 bp) AC186747
**SbRPG854** DQ185896	mitochondrial rieske protein	**2** Os04g32660.1 Os02g32120.1	2	2	5	1	MA4_111B14 (146821 bp) AC186954
**CIR257** DQ334868	GA-20 oxidase	-	-	-	6	1	MA4_82I11 (102232 bp) AC186955
						-	MBP_81C12 (142973 bp) AC186754
**CIR560** DQ334869	beta 1–3 glucanase	-	-	-	21	1	MA4_54N07 (96443 bp) AC186751
						-	MBP_91N22 (154246 bp) AC186755

*AC number : accession number.

One BAC clone was selected for sequencing for probe SbRPG132. For probes SbRPG373, SbRPG661 and SbRPG851, which were found to be present in one or two copies in rice, two *Musa *BACs with distinct HindIII fingerprints that might be derived from homeologous regions were selected for sequencing with the aim of studying the evolution of lineage-specific duplications in both *Musa *and rice (Table 1). Two BAC clones from *M. acuminata *cv. Calcutta-4 (*Musa *A) and two BACs from *M. balbisiana *cv. PKW (*Musa *B) isolated using the genetically-mapped RFLP single-copy probes CIR560 and CIR257 [23] were also fully sequenced with the objective of studying the extent of synteny between *Musa *A and B species as well as against the rice genome. These RFLP probes were selected because they corresponded to genomic clones encoding genes of known function, CIR257 for a GA-20 oxidase and CIR560 for a beta 1–3 glucanase, previously shown to be associated to traits of agronomic importance in controlling plant height [45,46] and stress response [47-50], respectively.

### Analysis of 1.8 Mb of *Musa *genomic sequences reveals particular features for the *Musa *genes

#### Musa genome statistics

**Figure 2:**
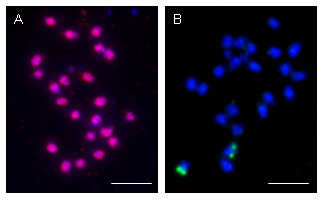
**Chromosome preparations of *M. acuminata *cv. Calcutta-4 (2n = 22) stained with DAPI after FISH of BAC**. (A) MA4_78I12 (detected with Texas red). (B) MA4_54N07 (detected FITC). Scale bar = 10 microns.

A total of 13 BACs (Table 1) were sequenced, generating over 1.4 Mb of unique *Musa *sequence. In order to provide a uniform set, data four additional BAC sequences (see Additional file 2 and [43]) were included in our annotation pipeline. These analyses revealed 443 predicted genes (on a total of 1.8 Mb of *Musa *genomic sequence from 17 BAC inserts), after elimination of all putative protein coding genes smaller than 100 amino acid residues. Approximately half of the gene models had matches in GenBank. Their classification based on similarities to genes found in the public sequence databases is presented in Additional file 3 and an annotation overview of the *Musa *genes in Additional file 4. Gene models were also compared against the *Musa *EST database donated to the Global *Musa *Genomics Consortium by Syngenta and maintained at the MIPS (Munich Information Center for Protein Sequences, Munich Germany), revealing that at least 10% of the predicted genes had a perfect match with EST sequences, thus probably being expressed in *Musa *tissues. Analysis of gene size, exon-intron structure and base composition for these 443 predicted genes is summarized in Table 2. The annotation revealed that, with the exception of MA4_78I12, the BACs analyzed were gene-rich (an average density of one gene per 4.1 kb). Our annotation of MuH9 revealed a total of 23 gene models for an average gene density of one gene per 3.6 kb compared with one gene per 6.9 kb based upon the earlier annotation [43]. In the case of MuG9, our pipeline predicted a total of 14 gene models in the first 52 kb of this BAC followed by a region of ~21 kb containing only transposons (5). Thus the gene density in the non-transposon containing region is one gene per 3.7 kb, very similar to MuH9. Previous annotation of this BAC [43] predicted 7 genes in the same 52 kb region for a density of one gene per 7 kb, with the remainder being transposon-related. The difference between the two annotations is due mainly to the larger number of hypothetical genes identified by the TIGR pipeline as well as some gene splits (e.g. MuH9-5 is split into three genes). Like the last ~21 kb of MuG9, BAC MA4_78I12 found to be mainly composed of class II transposable elements and also contains 7 interspersed predicted genes of which only the homolog of the SbRPG661 probe had a match in public databases. BAC-FISH experiments showed that BAC MA4_78I12 hybridized to all *M. acuminata *chromosomes except their extremities (Figure 2A). This pattern of hybridization is similar to what we observed by genomic *in situ *hybridization (GISH) using total genomic DNA as a probe and suggested that the extremities of the chromosomes are poor in repeated sequences [14]. Two gene-rich BACs (MA4_54N07 and MA4_82I11) were also analyzed by BAC-FISH and each hybridized at the extremity of one chromosome (see Figure 2B for MA4_54N07).

**Table 2:** Features of *Musa *genes in comparison with those of Arabidopsis and rice.

	***Musa***^1^	**Arabidopsis**^2^	**Rice**^3^
**GC content: overall (%)**	39.0	36.0	43.5
**Exons (%)**	48.4	44.2	53.1
**Introns (%)**	38.4	32.3	38.7
**Intergenic (%)**	38.5	31.2	41.4
**Exon length (bp)**	252	276	312
**Intron length (bp)**	366	169	364
**Number of exons/gene**	4.8	5.4	4.2
**Gene length (bp)**	2,504	2,232	2,519
**Protein length (aa)**	411	417	437
**Gene density (kb/gene)**	4.1	4.5	6.2

^1^Based on 1.8 Mb of genomic sequences

^2^[18]

^3^[120]

#### Base Composition and GC Distribution along the Musa genes

**Figure 3:**
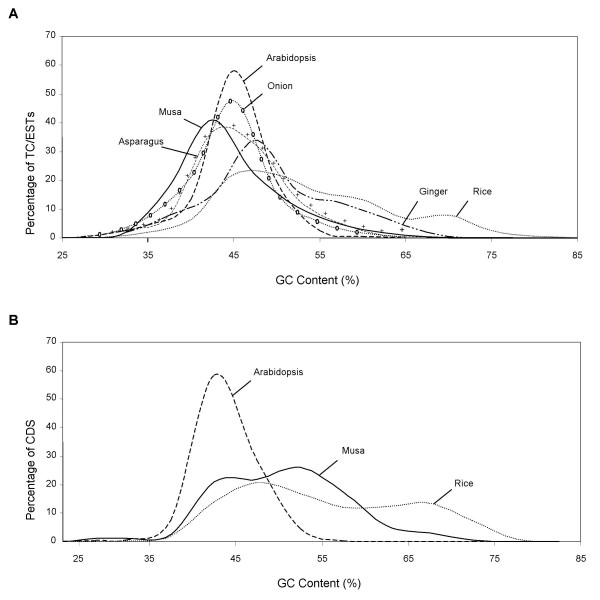
**Distribution of GC content in *Musa *and its comparison with other plant species**. (A) All TCs/ESTs from the named species. (B) All annotated CDS from 17 *Musa *BACs (this data set) and the complete genomes of Arabidopsis and rice.

**Figure 4:**
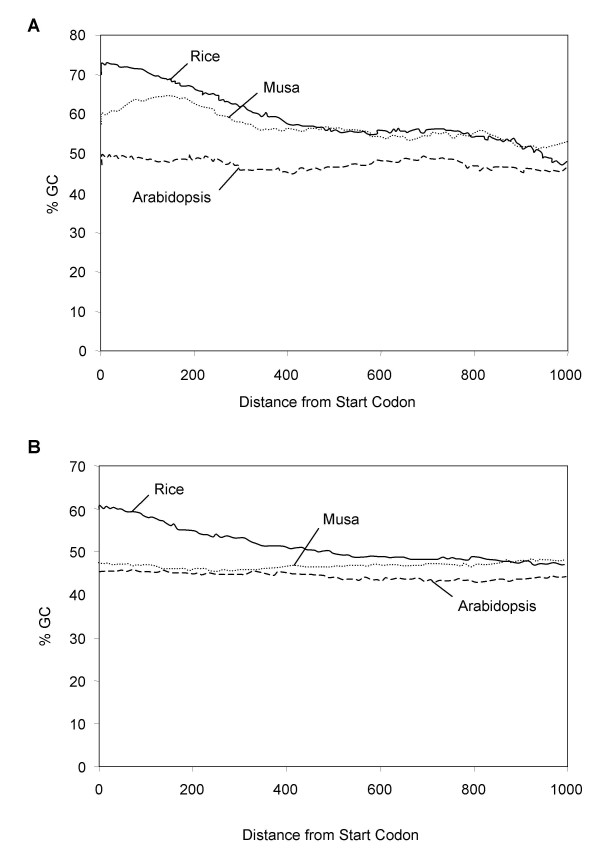
**Mean GC content from 5' to 3' across 129 bp sliding windows**. (A) for 77 *Musa *genes with a "rice-like" gradient. (B) for 180 *Musa *genes with an "Aradidopsis-like" pattern

The GC content of *Musa *coding sequences was compared with those of other monocots (rice, onion, asparagus) and dicots (Arabidopsis) using two data sets -unigene clusters and -singleton ESTs found in the TIGR plant transcript assembly database (TC/ESTs; [51]) and the 443 annotated genes (CDS) from the 17 *Musa *spp. sequenced BAC clones (Figure 3). The GC distributions of TC/EST (Figure 3A) and CDS regions of the *Musa *BACs (Figure 3B) were found to be asymmetrical and bimodal as compared to Arabidopsis and onion which are clearly symmetrical and unimodal (this report, [52,53]). The *Musa *GC content distribution resembles that of rice and other Poales with higher average GC content than eudicots (see also Table 2) and a long tail towards high GC values. We next examined GC content along the direction of transcription from the ATG start codon for each predicted *Musa *CDS using a sliding window of 129 bases (Figure 4). By manual inspection of the data, we were able to identify two categories of GC profiles from the *Musa *CDS: the first set shows a marked "rice-like" gradient of GC composition from 5' to 3' end and a higher GC content than Arabidopsis all along the CDS (Figure 4A), and the second set is "Arabidopsis-like" lacking a significant GC gradient from 5' to 3' (Figure 4B).

### Analysis of *Musa *repetitive elements

Several approaches were used to characterize the genomic sequence with respect to repeats. Database searches of the predicted genes against a non-redundant protein database (see Methods section) revealed a total of 78 transposable element (TE)-related sequences. Excluding TE-rich BAC MA4_78I12, there are on average ~2.6 retrotransposons (TE of class I) per 100 kb. Only one TE of class II encoded protein was detected. BAC sequences were also screened for previously characterized *Musa *RADKA repeats [15]; an average of 1.8 RADKA-related repeats (GenBank Accessions AF399938-AF399941, AF399943-AF399946 and AF399948) per 100 kb were identified. In an attempt to identify as yet uncharacterized repeats, the BAC sequences were also analyzed by RepeatScout [54]. After removing repeats having similarity to Arabidopsis or rice proteins, *Musa *CDS, RADKA sequences and transposable elements, six repeats with at least three copies were identified (data not shown). Five of these sequences have no significant hits to genes in GenBank while the sixth matches GenBank accession X99496 with a strong similarity to a part of the *Musa ycf2 *chloroplast gene. Analysis of individual BACs with PrintRepeats [55] shows that each BAC contains only a small number of regions that are repeated within the BAC, an observation that is supported by the relative ease with which the BAC sequences could be closed and finished.

### Microsynteny analysis between *Musa *and either rice or Arabidopsis

The 443 *Musa *predicted proteins were aligned against the rice and Arabidopsis proteomes. The results showed that 268 and 224 *Musa *proteins have hits with an E-value threshold of 1e-10 against the rice and Arabidopsis proteomes, respectively. The relative positions of the homologous genes identified in the rice and Arabidopsis genomes were compared to the order of the corresponding *Musa *genes with i-ADHoRe software [56]. Using this stringent approach, we were able to identify nine *Musa *BAC sequences showing microsynteny among the 17 *Musa *BACs analyzed: eight cases with rice and one case with Arabidopsis (Additional file 5).

**Figure 5:**
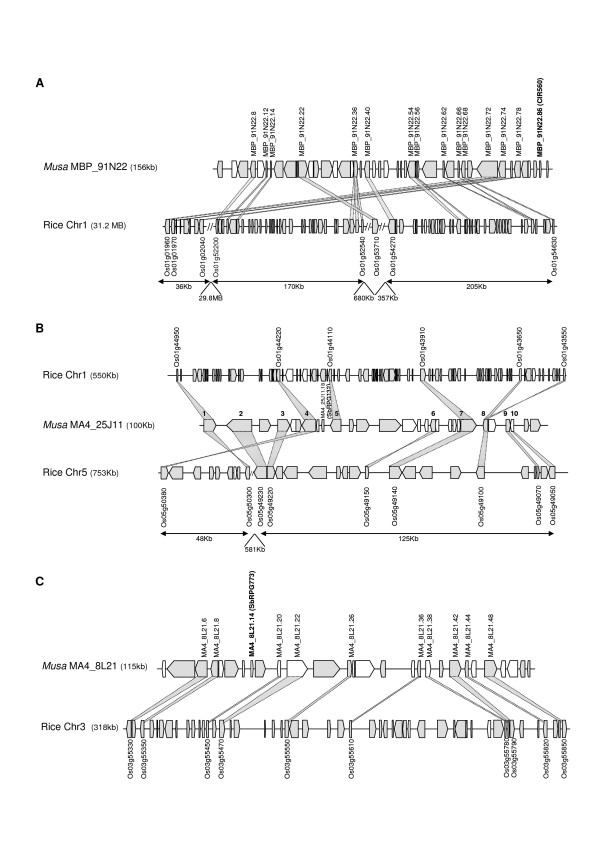
***Musa*-rice syntenic regions**. Predicted genes and their orientation are shown as boxed areas. Genes annotated such as hypothetical genes are represented in white. The probes used to identify the *Musa *BAC clones are indicated in brackets. Conserved genes between *Musa *and rice regions are connected by shaded areas. (A) Syntenic relationship between *Musa *MBP_91N22 BAC clone and rice chromosome 1. (B) Syntenic relationship between *Musa *MA4_25J11 BAC clone and rice chromosome 1 and 5. The numbers above the genes correspond to the locus numbers used for phylogenetic analyses. (C) Syntenic relationship between *Musa *MA4_8L21 BAC clone and rice chromosome 3.

The i-ADHoRe analyses identified syntenic blocks of three to ten genes (Additional file 5). We then refined the analyses by conducting reciprocal BLASTP searches between the genes in the orthologous regions. This analysis extended the number of genes included in these syntenic blocks. The most interesting cases of synteny conservation were found between BAC MBP_91N22 and rice chromosome 1 (Figure 5A), BAC MA4_25J11 and rice chromosomes 1 and 5 (Figure 5B), BAC MA4_8L21 and rice chromosome 3 (Figure 5C), BAC MuH9 and rice chromosome 4 (Additional file 6), and BAC MA4_42M13 and rice chromosome 2 (Additional file 7). Between five and eleven genes were found in common between the syntenic *Musa *and rice orthologous regions. In most cases the common genes were found in the same order and orientation in rice and *Musa*. However, several additional genes were typically found between the shared orthologs. Interestingly, the number of genes without orthologs within otherwise syntenic regions is much higher in rice as compared to *Musa*. This could be explained by differences between the rice and *Musa *lineages in the rate of translocation, duplication and gene death. Note also that *Musa *BAC MBP_91N22 displays conservation of synteny with two very distant segments on rice chromosome 1.

**Figure 6:**
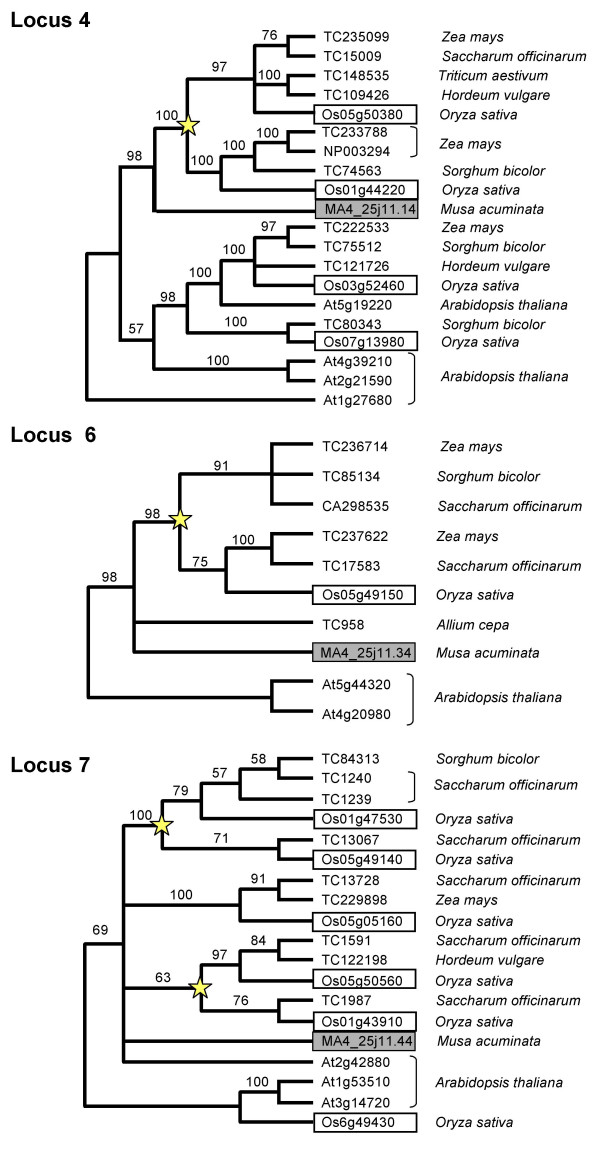
**Phylogenetic analyses on three of the ten *M. acuminata *genes from MA4_25J11 BAC clone**. These three *Musa *genes have homologous genes in rice chromosomes 1 and 5 and the locus numbers are taken from Figure 5B. Stars indicate duplication events in the most recent common ancestor of major grain lineages (i.e. rice, wheat and maize). MA4_25J11 BAC clone was isolated by the SbRPG132 probe.

In the case of BAC MA4_25J11, two rice orthologous regions were found with i-ADHoRe analyses and reciprocal BLASTP searches (rice chromosomes 1 and 5), revealing a duplication of this region in the rice genome. It is interesting to note that the two rice orthologous regions on chromosomes 1 and 5 have lost different sets of genes compared to *Musa*, as has been observed previously in other duplicated regions in angiosperms [37,57,58]. Phylogenetic analyses on the 10 *Musa *genes from BAC MA4_25J11 and co-orthologs [59] found on rice chromosomes 1 and 5 revealed that these regions were the product of the genome-wide duplication that has been hypothesized to have occurred early in the history of the Poaceae [56,60-62]. Duplicate maize, sugarcane, *Sorghum*, wheat, and barley genes occur in two separate clades in trees for loci 4, 6 and 7 (Figure 6 and Additional file 8) indicating that the duplication occurred before the divergence of the major grain lineages including rice, maize and wheat.

The only significant case of microcolinearity found between *Musa *and Arabidopsis involved three consecutive genes (Additional file 9). Interestingly, this *Musa-*Arabidopsis syntenic block was not found to be conserved in rice.

### Syntenic relationships between two regions of *M. acuminata *and *M. balbisiana*

**Figure 7:**
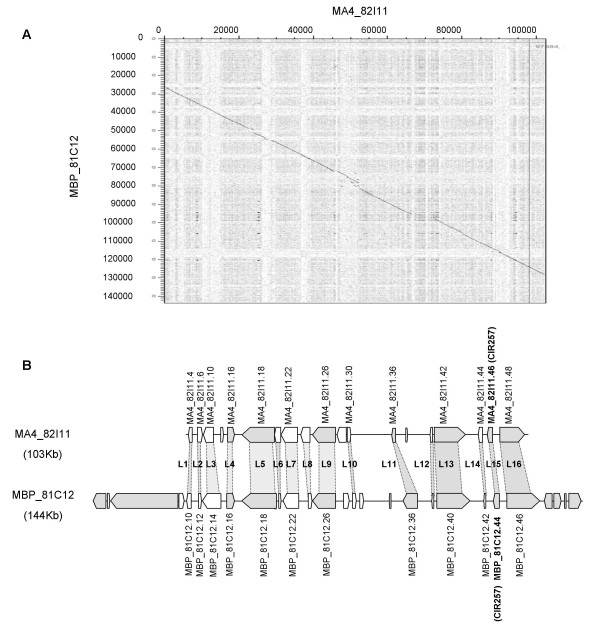
**Comparison between *M. acuminata *MA4_82I11 BAC clone and *M. balbisiana *MBP_81C12 BAC clone**. Predicted genes and their orientation are shown as boxed areas. Genes annotated such as hypothetical genes are represented in white. The probe used to identify the *Musa *BAC clones is indicated in brackets. Conserved genes between the two *Musa *regions are connected by shaded areas. (A) Dot plot analysis of the two pairs of homeologous BACs from *M. acuminata *and *M. balbisiana*.(B) Diagram of the syntenic regions between the two BAC clones.

We also investigated conservation of synteny between two regions of the genomes of *M. acuminata *and *M. balbisiana *species. Hybrids between these two species represent the majority of cultivated *Musa *genotypes worldwide. To carry out this pilot study, we selected BACs from orthologous regions of two single-copy, genetically-mapped RFLP probes (CIR560 and CIR257) encoding genes of agronomic interest. In both cases a high level of sequence conservation was found (see Figure 7 and Additional file 10) over the entire length of the sequenced regions in common between the two pairs of BACs analyzed (82.9% of nucleotide sequence identity for the MA4_82I11- MBP_81C12 pair and 87.6 % for the MA4_54N7- MBP_91N22 pair). The overall levels of sequence identity in genic regions were similar between the two pairs of orthologous BACs: 96.0 % for the MA4_82I11- MBP_81C12 pair and 96.3 % for the MA4_54N7- MBP_91N22 pair (based on the aligned orthologous gene pairs defined in Table 3; see the following paragraph for further details). A high degree of synteny was found between the orthologous sequences in both gene content and gene orientation. However, we observed some incongruence between the gene predictions of the orthologous BACs whose protein products have no match in public databases (i.e. hypothetical protein genes). In contrast, the predicted structures of genes that are homologous to known sequences were largely congruent between the orthologous BACs. Given the high levels of sequence conservation between the two *Musa *species, such variation of gene structure and exon/intron boundaries is unlikely for most functional genes. Hence, this analysis supports that further validation of gene models through additional EST sequencing or targeted RT-PCR is required.

**Table 3:** Level of synonymous substitution (K_s_) between homologous sequences in *M. acuminata *and *M. balbisiana*.

**Locus**	**Length**	**K**_s_	**Predicted function**
L4	1065	0.0496	GDSL-motif lipase/hydrolase family protein
L5	1401	0.0311	protein kinase family protein
L7	1341	0.0318	hypothetical protein
L9	1647	0.0252	protein kinase family protein
L11	825	0.0323	protein kinase-related
L13	2901	0.0420	leucine-rich repeat-containing protein kinase family protein
L15	1140	0.0435	gibberellin 20-oxidase family protein
L16	1899	0.0342	glucose-inhibited division A family protein
L18 *	1371	0.0960	leucine rich repeat family protein
L20	4119	0.0231	transcriptional repressor protein-related
L23	1941	0.0369	protein kinase family protein
L24	1737	0.0413	exostosin family protein
L26	2145	0.0461	kinesin light chain-related
K_s _mean	23532	**0.0410**	
Concatenation K_s_	23532	**0.0349**	

* Alignments between the *M. acuminata *gene and the orthologous sequence on the *M. balbisiana *sequence were identified by BLAST

### Divergence between *M. acuminata *and *M. balbisiana*

In order to evaluate the degree of divergence between the two *Musa *genomes, we obtained maximum likelihood estimates for K_s _values comparing pairs of orthologous genes identified in the *M. acuminata *and *M. balbisiana *BACs. We restricted our analysis to those genes (detailed in Table 3) that were intact and matched known gene sequences. For example the gene model for the 14^th ^locus in the *M. acuminata *genome (Figure 7; L14) is similar to a pectinesterase related protein, but the gene model was excluded from the analysis because the predicted coding sequence contained several in-frame stop codons indicating that this sequence is a pseudogene. The estimated K_s _values ranged from 0.0231 (Additional file 10; L19) to 0.0960 (L17), far below saturation levels (i.e. K_s _<< 1), with an average of 0.0410 (Table 3). Applying an average synonymous substitution rate of 4.5 per 10^9 ^years for nuclear genes in the Zingiberales (see below), this suggests that *M. acuminata *and *M. balbisiana *diverged approximately 4.6 Mya ago.

### Evidence for a large-scale duplication event in the *Musa *ancestor

**Figure 8:**
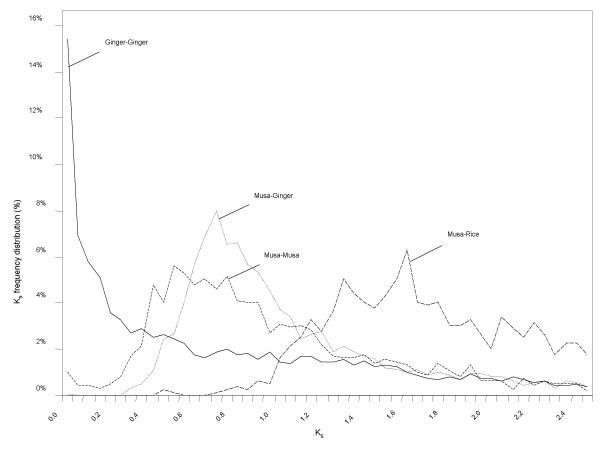
**Frequency of synonymous substitution (K_s_) in different pairwise species comparisons**. These results reveal the existence of whole genome duplications within *Musa *and revealed an extensive event pre-dating the ginger-*Musa *or rice-*Musa *divergences.

We also estimated the K_s _values between 1,446 pairs of duplicated *Musa *genes identified among 15,661 EST-derived unigenes found to be part of the paralog sets [63]. The distribution of K_s _values was estimated in order to assess spikes in the accumulation of duplicated genes [64]. If we assume that gene duplications and gene deletions are random and have relatively steady rates during the course of evolution, such a distribution is expected to show an L shape [64-68]. The distribution of K_s _values for duplicated *Musa *genes exhibits a large peak centered around K_s _= 0.55 (Figure 8) indicating an increase in the number of gene duplications that occurred in the *Musa *ancestor *circa *61 Mya (assuming a synonymous substitution rate of 4.5 per 10^9 ^years; see below). This ancient burst of duplications is likely the result of one or more large-scale duplication events. Alternatively, the observed duplications could be associated with a burst of transposon activity as has been hypothesized for some duplicate gene pairs in Arabidopsis [69]. However, analyses of K_s _plots for duplicated rice genes were unable to detect the 60 Mya duplication event in the Poacae that is evident in analyses of gene trees and duplicated blocks in the rice genome (e.g. [70,61]; this study). This may be due in part to the slower substitution rate we estimate for the Zingiberales relative to the Poaceae (see below).

We also analyzed the 18,612 ginger (*Zingiber officinale*; Zingiberaceae, Zingiberales) EST-derived unigenes available on the TIGR Plant Transcript Assemblies web site [71] (sequences generated by David Gang, University of Arizona) and found no evidence of large-scale duplication in the K_s _distribution for paralogous pairs (Figure 8). Moreover, the modal K_s _for reciprocal best matches between the *Musa *and *Zingiber *unigene sets is 0.78 (Figure 8), larger than the mode for *Musa *paralogous pairs. The age of the most recent common ancestor for the Musacaceae and Zingiberaceae is estimated at 87 Mya [3,72,73]. This implies an average synonymous substitution rate of 4.5 per 10^9 ^years (0.78 synonymous substitutions per site/(2*87,000,000 years)), intermediate between rates estimated for the Poaceae (6.1–6.5 per 10^9 ^years) and palms in the order Arecales (2.61 per 10^9 ^years; [74]. We must emphasize that all of these rate estimates are approximate, based on rough estimates of minimum divergence times. However, regardless of ambiguities in substitution rate calibrations, our results indicate that the predicted large-scale duplication that occurred in the *Musa *lineage (K_s _= 0.55) post-dates the divergence of lineages leading to *Zingiber *and *Musa *(K_s _= 0.78), but occurred well before the separation of *Musa *A and *Musa *B (K_s _= 0.0410).

K_s _values were also computed on 1,034 pairs of homologous genes identified between the *Musa *ESTs and the rice genome sequences. As expected, the distribution of K_s _values between rice-*Musa *homologs form a single peak centred around K_s _= 1.7 (Figure 8). Using this K_s _value to estimate the age of the Poales-Zingiberales split is less straightforward than described above for the *Musa*-*Zingiber *split, because synonymous substitution rates clearly vary between these Commelinid monocot lineages.

BAC fingerprint analyses revealed that whereas SbRPG854 hybridized to a single locus in the *Musa *genome, SbRPG probes SbRPG132 hybridized to 6 regions, SbRPG663 hybridized to 5 loci, and two loci were identified for SbRPG373, SbRPG661 and SbRPG851 (Table 1 and Additional file 1). BACs representing both distinct loci hybridizing to probes SbRPG661, SbRPG373 and SbRPG851 were sequenced with the aim of dating the time of duplication relative to the divergence of the *Musa *and rice lineages. Pair-wise estimations of K_s_, the number of synonymous substitutions per synonymous site, were 0.93 (± 0.25), 1.39 (± 0.19) and 1.43 (± 0.60) for *Musa *homologs of the coding regions of SbRPG661 (thioredoxin), SbRPG851 (phosphoglycerate kinase) and SbRPG373 (hypothetical protein), respectively. Phylogenetic analyses suggested that the SbRPG851 *Musa *homologs duplicated prior to the divergence of the Poales and the Zingiberales, (probably independent from the large-scale duplication described above), and the SbRPG661 and SbRPG373 *Musa *homologs are sister to each other in the gene tree, suggesting the duplications arose after the divergence of the Poales and the Zingiberales (data not shown).

We also analyzed the degree of conservation between genomic regions surrounding SbRPG661, SbRPG851 and SbRPG373 duplicated genes in *Musa *and rice and found no synteny in regions anchored by these homologs. This absence of synteny could be explained by duplication events and subsequent gene losses or by the translocation of the focal genes.

## Discussion

### Analysis of *Musa *genes reveals some particular features

Sequencing and annotation of ~1.8 Mb of *Musa *genomic sequence indicated that most of the BACs analyzed were gene rich with a low content of transposable element. Our analyses of 443 *Musa *genes predicted revealed that *Musa *genes generally have a "rice-like" bimodal GC distribution with a very asymmetrical and long tail towards high GC content as in previous studies [43,44]. However, a second class of "Arabidopsis-like" genes was found with an overall low GC content and no significant gradient along the coding sequence. In contrast to a previous comparison of grass and non-grass monocots [52,53], our analyses suggest that Zingeberales genes share some characteristics with the genomes of both eudicots and members of the Poaceae. This result suggests that the *Musa *genome is more similar to cereal genomes relative to onion, asparagus and the basal-most monocot lineage, *Acorus*.

### Syntenic relationships between distantly-related monocots

Whereas widespread conservation of synteny has been well established for members of the grass family (Poaceae), gene order has not been generally conserved between rice and Arabidopsis (e.g. reviewed by [75]. Few studies have compared genome structure between the members of the Poaceae and other monocot families, but recent comparisons between onion, garden asparagus and rice have failed to find evidence of conservation of macro- or micro-synteny [76,77]. However the genomic tag approach developed by [78] has allowed detecting anchor points between grasses and monocots. In this study we were able to identify microsyntenic regions in the *Musa *and rice genomes that have persisted over some 117 million years of evolution since these two lineages diverged [2]. However, in all syntenic regions detected, the shared genes were separated by intervening genes reflecting the occurrence of numerous insertions and deletion of genes in both rice and *Musa*. Insertions and deletions have been observed between rice and Arabidopsis regions showing micro-colinearity [58] and to a much lower extent between colinear regions among Poacea genomes [37,79]. Further sequencing of the *Musa *and other monocot genomes will provide more insight on the extent of lineage-specific gene gain and loss in otherwise syntenic regions.

### A first insight into syntenic relationships between *Musa *A and B

We focused our pilot study on two genomic regions containing genes of agronomic importance for *Musa *and rice to gain insight into the extent of conservation between the two cultivated species, *M. acuminata *(A genome) and *M. balbisiana *(B genome). Our data revealed an extremely high level of colinearity between the two *Musa *genomes in both regions. However several insertions and deletions occurred during the period of divergence (~4.6 Mya) of the two *Musa *species. The high level of microsynteny between the two genomes is likely to accelerate gene isolation in *M. balbisiana *once the construction of the whole genome physical map of *M. acuminata *has been completed by the Global *Musa *Genomics Consortium.

### Unveiling the paleopolyploid nature of *Musa *species

There is accumulating data supporting that polyploidy is one of the most important evolutionary mechanisms influencing the structure and content of angiosperm genomes [80]. Our work indicates ancient polyploidization in the lineage leading to *Musa *approximately 60 Mya. Similar lineage-specific events were described in the Poaceae [81,82], Brassicaceae [56,83,84], *Populus *[31], Solanaceae, Leguminoceae [64], Papaveraceae, *Acorus*, the Magnoliids and the Nymphaceae [65]. Polyploidy has clearly been an important source of genetic variation across the angiosperms as retained duplicate genes typically show divergent patterns of gene expression [85,86]. In *Musa*, as in other plant species, novel phenotypes can emerge from this genomic amalgam, including some with high visibility to natural selection, such as organ size and disease resistance.

Of particular interest is the "composite" nature of the duplicated rice regions relative to the syntenic *Musa *BAC MA4_25J11; different sets of genes were lost in rice chromosome 1 and 5, respectively as compared to *Musa*. This type of evolution is likely to reflect a dynamic of duplication [62] and independent evolution in both monocot lineages including recurrent cycles of genome duplication followed by diploidization. This phenomenon was also identified by [58] in their analysis of differential gene loss following duplication events in rice and Arabidopsis. Furthermore, our phylogenetic analyses of gene sets including the genes on *Musa *BAC MA4_25J11, rice orthologs and related genes found in the Arabidopsis genome and TIGR gene indices corroborate previous results suggesting that a genome-wide duplication in the common ancestor of all major cereal lineages is responsible for the large duplicated segments observed in the rice genome [61,62,87]. This finding illustrates how comparative analyses of distantly-related monocot species can complement studies on cereal genomes.

### Is rice a good model to study the structure and evolution of *Musa *genomes?

The use of rice as a reference species to accelerate map-based cloning projects by extrapolating marker position data and increasing marker density in targeted regions has a proven efficiency among cereal crops (e.g. barley, wheat, *Sorghum*), with a perceivable trend towards decreased efficiency when phylogenetic distance increases. Our analyses of the amount of microsynteny between rice and *Musa *suggest that there are cases in which predictions based upon microsynteny are useful but also this may not be general. In addition although our data showed that *Musa *genome is more similar to grain genomes relative to onion, asparagus and the basal monocot, *Acorus*, the differences observed confirmed that cereal genomes are not representative of all monocots [52,53,76,77]. This work also highlight that comparative analyses between distantly-related species such as rice and *Musa *are very important to improve our understanding of monocot genomes and more generally of angiosperms genome evolution.

## Conclusion

In conclusion, this study represents the first effort to investigate the existence and extent of microsynteny between rice and *Musa*, two-distantly related monocot species. Our analyses revealed a higher degree of synteny than has been reported for other comparisons between the rice and species outside of the grass family. In addition, we identified evidence for  an extensive microsynteny between the two *Musa *species representing the progenitors of most cultivated genotypes. In addition, we identified evidences for an ancient genome-scale duplication event in the lineage leading to *Musa *and highlighted the complexity of analyzing the structure and evolution of plant genomes following independent cycles of genome duplication and diploidization.

## Methods

### Selection of *Musa *BAC clones

Nine probes known from previous data to be conserved between rice, *Musa acuminata *cv. Madang, *Musa balbisiana *cv. PKW and Arabidopsis and revealing single or very low copy number locus were selected. These nine probes (SbRPG) correspond to *Sorghum *cDNA developed by Rustica Prograin Génétique and CIRAD [88]. These cDNAs and two Musa genomic probes CIR257 and CIR560 [20] were used to screen high density filters of the *M. acuminata *Calcutta-4 BAC library [25] according to standard protocols [89](see Table 1). The probes CIR257 and CIR560 were also used to screen *M. balbisiana *cv. PKW BAC library [26]. BAC DNA of positive clones was isolated using a Qiagen Robot 9600 and Qiagen 96-well BAC DNA isolation kit and digested with the restriction enzyme HindIII. The HindIII fingerprints were then hybridized with the corresponding probe to determine the number of loci.

### BAC-FISH analysis

Chromosome preparations were made as described in D'Hont et al [14] from root tips of *M. acuminata *cv. Calcutta-4 cultivated in glasshouse. Fluorescent *in situ *hybridizations (FISH) were performed as described in D'Hont et al [14], with 30 ng of BAC DNA labeled with digoxigenin or biotin as probes and 50 ng/μl of sheared salmon sperm DNA. The chromosomes were counterstained with DAPI (4'.6-diamidino-2-phenylindole).

### BAC sequencing

Selected BAC clones were sequenced by similar shotgun approaches at The Institute for Genomic Research (TIGR), Empresa Brasileira de Pesquisa Agropecuária (EMBRAPA-CENARGEN), Universidade Catolica de Brasilia (UCB) and National Institute of Agrobiological Sciences (NIAS). At TIGR, purified BAC DNA was sheared by nebulization, size-selected (2–3 kb) and ligated into a pUC-derived vector, pHOS1, using BstXI linkers. BAC DNA sent for sequencing to EMBRAPA and UCB was fragmented at Genoscope Centre (Evry, Paris, France) using a hydroshearer, size-selected (5 kb) and ligated into pcDNA2.1 vector using BstXI linkers. Clones were sequenced from both ends using ABI Big Dye terminator chemistry on ABI 3730 sequencing machines at TIGR and using a DYEnamicTM ET Terminator Sequencing Kit (Amersham Pharmacia Biotech) on Applied Biosystems 377 sequencers at EMBRAPA and UCB. Sequences were assembled using TIGR assembler and additional directed sequencing reactions performed as necessary to complete the sequence to high quality. BAC shotgun sequencing from NIAS were performed using shotgun (2 kb and 5–7 kb) clones of 10x coverage and Big Dye Terminator Kit (ABI) on ABI 3700 sequencers, assembled with Phred/phrap software [90,91], and contig gaps were filled if necessary.

### Sequence annotation

Annotation of the BAC assemblies was carried out using the TIGR annotation pipeline, a collection of software known as Eukaryotic Genome Control (EGC) that serves as the central data management system. Each BAC sequence was processed through a series of algorithms for predicting genes (Genscan+, Genemark.hmm, Glimmer) [92-94], splice sites [95,96] and tRNAs [97]. The AAT package [98] was used for homology search against nucleotide and protein databases, that include plant-specific cDNA and EST sequences, TIGR plant gene indices [99], a non-redundant amino acid database filtered from public sources, and SwissProt [100]. Protein models generated by the searches and predictions are further searched against Markov model (HMM) databases, including PFAM [101], and automatically assigned a putative name based on domain hits or homology to previously identified proteins. Gene structures and names were manually inspected and refined as necessary. Annotated gene models were scanned for *Musa *transposable element nucleotide sequences downloaded from GenBank and then compared to a curated database of transposable element-encoded proteins [102]. The top match from each hit was used to classify the transposable element.

### Comparison of BACs with one another

In order to determine whether the BACs selected by hybridization actually arose from duplicated regions of the *M. acuminata *(A) genome or homeologous regions of the *M. balbisiana *(B) genome, or to identify duplicated regions in the *M. acuminata *(A) genome (pairs of BACs hybridizing with the same probes), each BAC was compared against all other BACs using MUMmer [103]; Dotter [104]; [105] and an all-by-all BLASTP search [106]. The sequence identity of the overlapping sequences between BACs: MA4_82I11 and MBP_81C12 or MA4_54N7 and MBP_91N22, was computed with Stretcher from the EMBOSS package [107].

### Synteny search

The 443 *Musa *predicted proteins were aligned against the rice and Arabidopsis proteomes using the BLASTP program (e-value < 1e-10) [108]. The i-ADHoRe software [56] which looks for regions where the gene order is similar between two genomic sequences was used with the following parameters: a gap size and a cluster gap of 40-40, a q value of 0.9, three anchor points and a probability cutoff of 0.001. For four BACs (MA4_25J11, MA4_8L21, MBP_91N22 and MuH9), we tried to extend the regions of synteny between *Musa *and rice found by i-ADHoRe by conducting reciprocal BLASTP searches between the genes corresponding on the homologous regions.

### Phylogenetic analyses

*Musa *genes were used in BLASTX searches to query a database of rice and Arabidopsis gene family clusters [109]. Translated blast searches (tBLASTX) against the TIGR plant gene indices [110] were also performed and inferred protein sequences with e-values < 1e-30 were compiled with homologous *Musa*, rice and Arabidopsis sequences. Amino acid alignments of the compiled sequences were constructed using MUSCLE [111] and manually adjusted. Parsimony analyses were performed on the amino acid alignments using PAUP* v4.0b10 [112].

### Contruction of unigenes

*Musa *EST sequences were provided by the Global *Musa *Genomics Consortium [113]. These sequences were first assembled into unigenes using the TGICL package [114] to eliminate sequence redundancy. Because unigenes are derived from EST sequences and so have no annotated open reading frames and may contain frameshift sequencing errors, the following approach was taken. Each unigene was aligned against the rice proteome (downloaded from GenBank) using BLASTX. The best match was considered significant if the alignment length was >100 amino acids and the Expect value (*E*) was <1e-15. The open reading frame was then extracted from the unigene sequence using the Genewise program (which can infer frameshift sites; [115] with the corresponding best match protein as a guide.

### Estimation of the level of synonymous substitution between two sequences

For each pair of coding sequence, the two translation products were aligned using the MUSCLE program [111] and the resulting alignment was used as a guide to align the nucleotide sequences. After removing gaps and N-containing codons, the level of synonymous substitution (K_s_) was estimated using the maximum likelihood method implemented in CODEML [116] under the F3x4 model [117].

### Distribution of the age of duplication of *Musa *genes

All-against-all nucleotide sequence similarity searches were done among the open reading frame extracted from the unigene sequences using BLASTN [106]. Sequences aligned over >300 bp and showing at least 40% identity were defined as pairs of paralogs. Then we estimated K_s _for each pair of paralogs. We systematically discarded one sequence from a pair of paralogs showing no synonymous substitutions (K_s _= 0) as well as all K_s _values involving this sequence to avoid the inclusion of redundant entries of the same gene in the analysis (see [64] for further details). A gene family of *n *members results from *n*-1 gene duplication events. However, the number of possible pairwise comparisons within a gene family (*n *× (*n*-1)/2) can be substantially larger than the number of gene duplications, which results in multiple estimates of the ages of some duplications. To eliminate the redundant K_s _values, pairs of duplicated sequences were grouped into gene families using a single linkage clustering method. Then we used the hierarchical clustering method described in [64] to reconstruct the approximated phylogeny of each gene family: (1) Initially, all sequences in the family were treated as a separate clusters. (2) Then, the K_s _values for all possible pairs of clusters were compared. (3) The pair of clusters having the smallest K_s _value was replaced by a single new cluster containing all their sequences. (4) The median K_s _value was chosen to represent the duplication event that gave rise to the two merged clusters. (5) Steps 2 to 4 were repeated until all sequences were contained in a single cluster. When two clusters A and B contained more than one sequence, their associated K_s _value in step 2 corresponded to the median K_s _obtained for all possible pairs of any sequence from A and any sequence from B.

## Abbreviations

aa – amino acid

AC number – accession number

CDS – coding DNA sequence

EST – expressed sequence tag

FISH – fluorescent *in situ *hybridization

GISH – genomic *in situ *hybridization

Ks – synonymous substitution rate

Mya – million years ago

PKW – Pisang Klutuk Wulung

RFLP – restriction fragment length polymorphism

TE – transposable element

TC – tentative consensi.

## Authors' contributions

PP, AD, GJP, TS, MTSJ, RNGM and JCG conceived of the study and participated in its design. PP, AYC, CMRS, OG, ADV, HK, TM, RA, TA, EH, GJP, RNGM and CDT performed the experiments. ML, MR, GB, JLM, FRDS, CMR, FC, BJH and CDT analysed and interpreted the data. RNGM contributed reagents. ML, GB, JLM, AD and CDT wrote the manuscript. All authors read and approved the final manuscript.

## Supplementary Material

Additional file 1 **Supplementary Table 1. Additional list of probes used to identify the *Musa *BAC clones**. Estimated copy numbers of these sequences in rice, *Sorghum *and *Musa *are indicated for SbRPG (*Sorghum bicolor*) sequences. Click here for file

Additional file 2 Supplementary Table 2. Additional BAC clones analyzed to define *Musa *gene features and syntenic relationships with rice. Click here for file

Additional file 3 Supplementary Table 3. Statistics of the 17 *Musa *BAC clones analyzed in the present study. Click here for file

Additional file 4 **Supplementary Table 4. Annotation overview of the *Musa *genes**. The column «Pseudogene» indicates by [1] if the gene is a pseudogene and [0] if not. Closest sequence homolog is the first similar protein sequence found by BLASTP after the sequence itself. Click here for file

Additional file 5 **Supplementary Table 5. List of genes involved in synteny relationship between *Musa *and rice based on i-ADHoRE results**. Multiplicon is a BAC genomic sequence on which the baseclusters are isolated and represents a cluster of 3 genes minima. Click here for file

Additional file 6 **Supplementary Figure 1. *Musa*-rice syntenic region between MuH9 BAC clone and rice chromosome 4**. Homologous genes between *Musa *and rice are indicated by shaded areas. Genes annotated such as hypothetical genes are white. Click here for file

Additional file 7 **Supplementary Figure 2. The *Musa*-rice syntenic region around the highly conserved porphobilinogen deaminase gene**. Shaded areas connect homologous genes conserved between chromosome 2 of rice and *Musa *MA4_42M13 BAC clone (isolated by SbRPG748 probe). Genes annotated such as hypothetical genes are white. In *Musa*, a recent local duplication of the porphobilinogen deaminase gene occurred (genes MA4_42M13.6 and MA4_42M13.8). Click here for file

Additional file 8 **Supplementary Figure 3. Phylogenetic analyses on the seven of the ten *M. acuminata *genes from MA4_25J11 BAC clone**. These seven *Musa *genes have homologous genes in rice chromosomes 1 and 5 and the locus numbers are available on Figure 5B. MA4_25J11 BAC clone was isolated by SbRPG132 probe. Click here for file

Additional file 9 **Supplementary Figure 4. *Musa*-Arabidopsis syntenic region between *Musa *MA4_54B05 BAC clone and Arabidopsis chromosome 5**. Homologous genes between *Musa *and Arabidopsis are indicated by shaded areas. Genes annotated such as hypothetical genes are white. MA4_54B05 BAC clone was isolated by SbRPG661 probe. Click here for file

Additional file 10 **Supplementary Figure 5. Collinearity between *M. acuminata *(MA4_54N07) and *M. balbisiana *(MBP_91N22) around the CIR560 marker**. The shaded areas connecting the two genomic regions represent conserved genes. Predicted genes and their orientation in each *Musa *BAC clone are shown as boxed areas. The genes for which the name is in bold hybridize with the marker. Genes annotated such as hypothetical genes are white. (A) Dot plot analysis of the two pairs of homeologous BACs from *M. acuminata *and *M. balbisiana*. (B) Diagram of the syntenic regions between the two BAC clones. Click here for file

## References

[B1] Arias P, Dankers C, Liu P, Pilkauskas P (2003). The world banana economy 1985–2002. FAO.

[B2] Janssen T, Bremer K (2004). The age of major monocot groups inferred from 800 + rbcL sequences. Botanical Journal of the Linnean Society.

[B3] Sanderson MJ, Thorne JL, Wikström N, Bremer K (2004). Molecular evidence on plant divergence times. American Journal of Botany.

[B4] Simmonds N, Shepherd K (1955). The taxonomy and origins of the cultivated bananas. Bot J Linn Soc.

[B5] Bartos J, Alkhimova O, Dolezelova M, De Langhe E, Dolezel J (2005). Nuclear genome size and genomic distribution of ribosomal DNA in Musa and Ensete (Musaceae): taxonomic implications. Cytogenet Genome Res.

[B6] Carreel F, Fauré S, Gonzalez de Leon D, Lagoda PJL, Perrier X, Bakry F, Tezenas du Montcel H, Lanaud C, Horry JP (1994). Evaluation of the genetic diversity in diploid bananas (Musa sp.). Genetics, Selection, Evolution.

[B7] Carreel F, Gonzalez de Leon D, Lagoda P, Lanaud C, Jenny C, Horry JP, Tezenas du Montcel H (2002). Ascertaining maternal and paternal lineage within Musa by chloroplast and mitochondrial DNA RFLP analyses. Genome.

[B8] Grapin A, Noyer JL, Dambier D, Carreel F, Lanaud C, Baurens F-C, Lagoda PJL (1998). Diploid Musa acuminata genetic diversity with Sequence Tagged Microsatellite Sites. Electrophoresis.

[B9] Noyer JL, Causse S, Tomekpe K, Bouet A, Baurens FC (2005). A new image of plantain diversity assessed by SSR, AFLP and MSAP markers. Genetica.

[B10] Raboin LM, Carreel F, Noyer J-L, Baurens F-C, Horry J-P, Bakry F, Tezenas Du Montcel H, Ganry J, Lanaud C, Lagoda PJL (2005). Diploid Ancestors of Triploid Export Banana Cultivars: Molecular Identification of 2n Restitution Gamete Donors and n Gamete Donors. Molecular breeding.

[B11] Ude G, Pillay M, Nwakanma D, Tenkouano A (2002). Genetic Diversity in Musa acuminata Colla and Musa balbisiana Colla and some of their natural hybrids using AFLP Markers. Theor Appl Genet.

[B12] Ge XJ, Liu MH, Wang K, Schaal BA, Chiang TY (2005). Population structure of wild bananas, Musa balbisiana, in China determined by SSR fingerprinting and cpDNA PCR-RFLP. Molecular ecology.

[B13] Baurens FC, Noyer JL, Lanaud C, Lagoda PJ (1996). Use of competitive PCR to assay copy number of repetitive elements in banana. Mol Gen Genet.

[B14] D'Hont A, Paget-Goy A, Escoute J, Carreel F (2000). The interspecific genome structure of cultivated banana, Musa spp. revealed by genomic DNA in situ hybridization. Theor Appl Genet.

[B15] Valarik M, Simkova H, Hribova E, Safar J, Dolezelova M, Dolezel J (2002). Isolation, characterization and chromosome localization of repetitive DNA sequences in bananas (Musa spp.). Chromosome Res.

[B16] Kamate K, Brown S, Durand P, Bureau JM, De Nay D, Trinh TH (2001). Nuclear DNA content and base composition in 28 taxa of Musa. Genome.

[B17] Lysak M, Dolezelova M, Horry J, Swennen R, Dolezel J (1999). Flow cytometric analysis of nuclear DNA content in Musa. Theor Appl Genet.

[B18] Arabidopsis Genome Initiative (2000). Analysis of the genome sequence of the flowering plant Arabidopsis thaliana. Nature.

[B19] International Rice Genome Sequencing Project (2005). The map-based sequence of the rice genome. Nature.

[B20] Fauré S, Noyer J, Horry J, Bakry F, Lanaud C, Gonzalez D, Leon D (1993). A molecular marker-based linkage map of diploid bananas (Musa acuminata). Theor Appl Genet.

[B21] Noyer J, Dambier D, Lanaud C, Lagoda P (1997). The saturated map of diploid banana (Musa acuminata). Abstract Plant & Animal Genome V Conference.

[B22] Vilarinhos A, Carreel F, Rodier M, Hippolyte I, Benabdelmouna A, Triaire D, Bakry F, Courtois B, D'Hont A (2006). Characterization Of Translocations In Banana By FISH Of BAC Clones Anchored To A Genetic Map. Plant & Animal Genomes XIV Conference.

[B23] Tropgenedb. http://tropgenedb.cirad.fr/en/banana.html.

[B24] Ortiz-Vazquez E, Kaemmer D, Zhang HB, Muth J, Rodriguez-Mendiola M, Arias-Castro C, James A (2005). Construction and characterization of a plant transformation-competent BIBAC library of the black Sigatoka-resistant banana Musa acuminata cv. Tuu Gia (AA). Theor Appl Genet.

[B25] Vilarinhos AD, Piffanelli P, Lagoda P, Thibivilliers S, Sabau X, Carreel F, D'Hont A (2003). Construction and characterization of a bacterial artificial chromosome library of banana (Musa acuminata Colla). Theor Appl Genet.

[B26] Safar J, Noa-Carrazana JC, Vrana J, Bartos J, Alkhimova O, Sabau X, Simkova H, Lheureux F, Caruana ML, Dolezel J (2004). Creation of a BAC resource to study the structure and evolution of the banana (Musa balbisiana) genome. Genome.

[B27] Devos KM (2005). Updating the 'crop circle'. Curr Opin Plant Biol.

[B28] Mudge J, Cannon SB, Kalo P, Oldroyd GE, Roe BA, Town CD, Young ND (2005). Highly syntenic regions in the genomes of soybean, Medicago truncatula, and Arabidopsis thaliana. BMC Plant Biol.

[B29] Town CD, Cheung F, Maiti R, Crabtree J, Haas BJ, Wortman JR, Hine EE, Althoff R, Arbogast TS, Tallon LJ (2006). Comparative Genomics of Brassica oleracea and Arabidopsis thaliana Reveal Gene Loss, Fragmentation, and Dispersal after Polyploidy. Plant Cell.

[B30] Zhu H, Choi HK, Cook DR, Shoemaker RC (2005). Bridging model and crop legumes through comparative genomics. Plant Physiol.

[B31] Tuskan GA, Difazio S, Jansson S, Bohlmann J, Grigoriev I, Hellsten U, Putnam N, Ralph S, Rombauts S, Salamov A (2006). The genome of black cottonwood, Populus trichocarpa (Torr. & Gray). Science.

[B32] Jackson S, Rounsley S, Purugganan M (2006). Comparative sequencing of plant genomes: choices to make. Plant Cell.

[B33] Bowers JE, Arias MA, Asher R, Avise JA, Ball RT, Brewer GA, Buss RW, Chen AH, Edwards TM, Estill JC (2005). Comparative physical mapping links conservation of microsynteny to chromosome structure and recombination in grasses. Proc Natl Acad Sci USA.

[B34] Buell CR, Yuan Q, Ouyang S, Liu J, Zhu W, Wang A, Maiti R, Haas B, Wortman J, Pertea M (2005). Sequence, annotation, and analysis of synteny between rice chromosome 3 and diverged grass species. Genome Res.

[B35] La Rota M, Sorrells ME (2004). Comparative DNA sequence analysis of mapped wheat ESTs reveals the complexity of genome relationships between rice and wheat. Funct Integr Genomics.

[B36] Singh NK, Raghuvanshi S, Srivastava SK, Gaur A, Pal AK, Dalal V, Singh A, Ghazi IA, Bhargav A, Yadav M (2004). Sequence analysis of the long arm of rice chromosome 11 for rice-wheat synteny. Funct Integr Genomics.

[B37] Ilic K, SanMiguel PJ, Bennetzen JL (2003). A complex history of rearrangement in an orthologous region of the maize, sorghum, and rice genomes. Proc Natl Acad Sci USA.

[B38] Gu Y, Coleman-Derr D, Kong X, Anderson O (2004). Rapid genome evolution revealed by comparative sequence analysis of orthologous regions from four triticeae genomes. Plant Physiol.

[B39] Jaillon O, Aury J, Brunet F, Petit J, Stange-Thomann N, Mauceli E, Bouneau L, Fischer C, Ozouf-Costaz C, Bernot A (2004). Genome duplication in the teleost fish Tetraodon nigroviridis reveals the early vertebrate proto-karyotype. Nature.

[B40] Jannoo N, Grivet L, Chantret N, Garsmeur O, Glaszmann JC, Arruda P, D'Hont A (2007). Orthologous comparison in a gene-rich region among grasses reveals stability in the sugarcane polyploid genome. Plant Journal.

[B41] Salse J, Piegu B, Cooke R, Delseny M (2002). Synteny between Arabidopsis thaliana and rice at the genome level: a tool to identify conservation in the ongoing rice genome sequencing project. Nucleic Acids Res.

[B42] Salse J, Piegu B, Cooke R, Delseny M (2004). New in silico insight into the synteny between rice (Oryza sativa L.) and maize (Zea mays L.) highlights reshuffling and identifies new duplications in the rice genome. Plant J.

[B43] Aert R, Sagi L, Volckaert G (2004). Gene content and density in banana (Musa acuminata) as revealed by genomic sequencing of BAC clones. Theor Appl Genet.

[B44] Cheung F, Town CD (2007). A BAC end view of the Musa acuminata genome. BMC Plant Biol.

[B45] Oikawa T, Koshioka M, Kojima K, Yoshida H, Kawata M (2004). A role of OsGA20ox1, encoding an isoform of gibberellin 20-oxidase, for regulation of plant stature in rice. Plant Mol Biol.

[B46] Spielmeyer W, Ellis MH, Chandler PM (2002). Semidwarf (sd-1), "green revolution" rice, contains a defective gibberellin 20-oxidase gene. Proc Natl Acad Sci USA.

[B47] Nishizawa Y, Saruta M, Nakazono K, Nishio Z, Soma M, Yoshida T, Nakajima E, Hibi T (2003). Characterization of transgenic rice plants over-expressing the stress-inducible beta-glucanase gene Gns1. Plant Mol Biol.

[B48] Thomas BR, Romero GO, Nevins DJ, Rodriguez RL (2000). New perspectives on the endo-beta-glucanases of glycosyl hydrolase Family 17. Int J Biol Macromol.

[B49] Romero GO, Simmons C, Yaneshita M, Doan M, Thomas BR, Rodriguez RL (1998). Characterization of rice endo-beta-glucanase genes (Gns2-Gns14) defines a new subgroup within the gene family. Gene.

[B50] Simmons CR, Litts JC, Huang N, Rodriguez RL (1992). Structure of a rice beta-glucanase gene regulated by ethylene, cytokinin, wounding, salicylic acid and fungal elicitors. Plant Mol Biol.

[B51] Childs KL, Hamilton JP, Zhu W, Ly E, Cheung F, Wu H, Rabinowicz PD, Town CD, Buell CR, Chan AP (2007). The TIGR Plant Transcript Assemblies database. Nucleic Acids Res.

[B52] Kuhl JC, Cheung F, Yuan Q, Martin W, Zewdie Y, McCallum J, Catanach A, Rutherford P, Sink KC, Jenderek M (2004). A unique set of 11,008 onion expressed sequence tags reveals expressed sequence and genomic differences between the monocot orders Asparagales and Poales. Plant Cell.

[B53] Kuhl JC, Havey MJ, Martin WJ, Cheung F, Yuan Q, Landherr L, Hu Y, Leebens-Mack J, Town CD, Sink KC (2005). Comparative genomic analyses in Asparagus. Genome.

[B54] Price AL, Jones NC, Pevzner PA (2005). De novo identification of repeat families in large genomes. Bioinformatics.

[B55] Parsons JD (1995). Miropeats: graphical DNA sequence comparisons. Comput Appl Biosci.

[B56] Simillion C, Vandepoele K, Saeys Y, Van de Peer Y (2004). Building genomic profiles for uncovering segmental homology in the twilight zone. Genome Res.

[B57] Sampedro J, Lee Y, Carey RE, dePamphilis C, Cosgrove DJ (2005). Use of genomic history to improve phylogeny and understanding of births and deaths in a gene family. Plant J.

[B58] Vandepoele K, Simillion C, Van de Peer Y (2002). Detecting the undetectable: uncovering duplicated segments in Arabidopsis by comparison with rice. Trends Genet.

[B59] Sonnhammer EL, Koonin EV (2002). Orthology, paralogy and proposed classification for paralog subtypes. Trends Genet.

[B60] Blanc G, Barakat A, Guyot R, Cooke R, Delseny M (2000). Extensive duplication and reshuffling in the Arabidopsis genome. Plant Cell.

[B61] Paterson AH, Bowers JE, Chapman BA (2004). Ancient polyploidization predating divergence of the cereals, and its consequences for comparative genomics. Proc Natl Acad Sci USA.

[B62] Yu J, Wang J, Lin W, Li S, Li H, Zhou J, Ni P, Dong W, Hu S, Zeng C (2005). The Genomes of Oryza sativa: a history of duplications. PLoS Biol.

[B63] A Global Programme for Musa Improvement. http://www.promusa.org/.

[B64] Blanc G, Wolfe KH (2004). Widespread paleopolyploidy in model plant species inferred from age distributions of duplicate genes. Plant Cell.

[B65] Cui L, Wall PK, Leebens-Mack JH, Lindsay BG, Soltis DE, Doyle JJ, Soltis PS, Carlson JE, Arumuganathan K, Barakat A (2006). Widespread genome duplications throughout the history of flowering plants. Genome Res.

[B66] Lynch M, Conery JS (2000). The evolutionary fate and consequences of duplicate genes. Science.

[B67] Maere S, De Bodt S, Raes J, Casneuf T, Van Montagu M, Kuiper M, Van de Peer Y (2005). Modeling gene and genome duplications in eukaryotes. Proc Natl Acad Sci USA.

[B68] Schlueter SD, Wilkerson MD, Huala E, Rhee SY, Brendel V (2005). Community-based gene structure annotation. Trends Plant Sci.

[B69] Hughes AL, Friedman R, Ekollu V, Rose JR (2003). Non-random association of transposable elements with duplicated genomic blocks in Arabidopsis thaliana. Mol Phylogenet Evol.

[B70] Vandepoele K, Simillion C, Van de Peer Y (2003). Evidence that rice and other cereals are ancient aneuploids. Plant Cell.

[B71] The TIGR Plant Transcript Assemblies database. http://plantta.tigr.org/.

[B72] Kress WJ, Prince LM, Hahn WJ, Zimmer EA (2001). Unraveling the evolutionary radiation of the families of the Zingiberales using morphological and molecular evidence. Syst Biol.

[B73] Bremer K (2000). Early Cretaceous lineages of monocot flowering plants. Proc Natl Acad Sci USA.

[B74] Gaut BS, Morton BR, McCaig BC, Clegg MT (1996). Substitution rate comparisons between grasses and palms: synonymous rate differences at the nuclear gene Adh parallel rate differences at the plastid gene rbcL. Proc Natl Acad Sci USA.

[B75] Bennetzen JL, Ma J, Devos KM (2005). Mechanisms of recent genome size variation in flowering plants. Ann Bot (Lond).

[B76] Martin WJ, McCallum J, Shigyo M, Jakse J, Kuhl JC, Yamane N, Pither-Joyce M, Gokce AF, Sink KC, Town CD (2005). Genetic mapping of expressed sequences in onion and in silico comparisons with rice show scant colinearity. Mol Genet Genomics.

[B77] Jakse J, Telgmann A, Jung C, Khar A, Melgar S, Cheung F, Town CD, Havey MJ (2006). Comparative sequence and genetic analyses of asparagus BACs reveal no microsynteny with onion or rice. Theor Appl Genet.

[B78] Lohithaswa HC, Feltus FA, Singh HP, Bacon CD, Bailey CD, Paterson AH (2007). Leveraging the rice genome sequence for monocot comparative and translational genomics. Theor Appl Genet.

[B79] Dubcovsky J, Ramakrishna W, SanMiguel PJ, Busso CS, Yan L, Shiloff BA, Bennetzen JL (2001). Comparative sequence analysis of colinear barley and rice bacterial artificial chromosomes. Plant Physiol.

[B80] Adams KL, Wendel JF (2005). Polyploidy and genome evolution in plants. Curr Opin Plant Biol.

[B81] Paterson AH, Bowers JE, Peterson DG, Estill JC, Chapman BA (2003). Structure and evolution of cereal genomes. Curr Opin Genet Dev.

[B82] Wang X, Shi X, Hao B, Ge S, Luo J (2005). Duplication and DNA segmental loss in the rice genome: implications for diploidization. New Phytol.

[B83] Vision TJ, Brown DG, Tanksley SD (2000). The origins of genomic duplications in Arabidopsis. Science.

[B84] Bowers JE, Abbey C, Anderson S, Chang C, Draye X, Hoppe AH, Jessup R, Lemke C, Lennington J, Li Z (2003). A high-density genetic recombination map of sequence-tagged sites for sorghum, as a framework for comparative structural and evolutionary genomics of tropical grains and grasses. Genetics.

[B85] Blanc G, Wolfe KH (2004). Functional divergence of duplicated genes formed by polyploidy during Arabidopsis evolution. Plant Cell.

[B86] Duarte JM, Cui L, Wall PK, Zhang Q, Zhang X, Leebens-Mack J, Ma H, Altman N, dePamphilis CW (2006). Expression pattern shifts following duplication indicative of subfunctionalization and neofunctionalization in regulatory genes of Arabidopsis. Mol Biol Evol.

[B87] Rong J, Abbey C, Bowers JE, Brubaker CL, Chang C, Chee PW, Delmonte TA, Ding X, Garza JJ, Marler BS (2004). A 3347-locus genetic recombination map of sequence-tagged sites reveals features of genome organization, transmission and evolution of cotton (Gossypium). Genetics.

[B88] Boivin K, Deu M, Rami J-F, Trouche G, Hamon P (1999). Towards a saturated sorghum map using RFLP and AFLP markers. Theor Appl Genet.

[B89] Luo M, Wang YH, Frisch D, Joobeur T, Wing RA, Dean RA (2001). Melon bacterial artificial chromosome (BAC) library construction using improved methods and identification of clones linked to the locus conferring resistance to melon Fusarium wilt (Fom-2). Genome.

[B90] Ewing B, Green P (1998). Base-calling of automated sequencer traces using phred. II. Error probabilities. Genome Res.

[B91] Ewing B, Hillier L, Wendl MC, Green P (1998). Base-calling of automated sequencer traces using phred. I. Accuracy assessment. Genome Res.

[B92] Burge C, Karlin S (1997). Prediction of complete gene structures in human genomic DNA. J Mol Biol.

[B93] Lukashin AV, Borodovsky M (1998). GeneMark.hmm: new solutions for gene finding. Nucleic Acids Res.

[B94] Salzberg SL, Pertea M, Delcher AL, Gardner MJ, Tettelin H (1999). Interpolated Markov models for eukaryotic gene finding. Genomics.

[B95] Pertea M, Lin X, Salzberg SL (2001). GeneSplicer: a new computational method for splice site prediction. Nucleic Acids Res.

[B96] Hebsgaard SM, Korning PG, Tolstrup N, Engelbrecht J, Rouze P, Brunak S (1996). Splice site prediction in Arabidopsis thaliana pre-mRNA by combining local and global sequence information. Nucleic Acids Res.

[B97] Lowe TM, Eddy SR (1997). tRNAscan-SE: a program for improved detection of transfer RNA genes in genomic sequence. Nucleic Acids Res.

[B98] Huang X, Adams MD, Zhou H, Kerlavage AR (1997). A tool for analyzing and annotating genomic sequences. Genomics.

[B99] Quackenbush J, Liang F, Holt I, Pertea G, Upton J (2000). The TIGR gene indices: reconstruction and representation of expressed gene sequences. Nucleic Acids Res.

[B100] Bairoch A, Apweiler R (2000). The SWISS-PROT protein sequence database and its supplement TrEMBL in 2000. Nucleic Acids Res.

[B101] Bateman A, Birney E, Cerruti L, Durbin R, Etwiller L, Eddy SR, Griffiths-Jones S, Howe KL, Marshall M, Sonnhammer EL (2002). The Pfam protein families database. Nucleic Acids Res.

[B102] Transposable elements database on TIGR FTP site. ftp://ftp.tigr.org/pub/data/TransposableElements/transposon_db.pep.

[B103] Delcher AL, Phillippy A, Carlton J, Salzberg SL (2002). Fast algorithms for large-scale genome alignment and comparison. Nucleic Acids Res.

[B104] Sonnhammer EL, Durbin R (1995). A dot-matrix program with dynamic threshold control suited for genomic DNA and protein sequence analysis. Gene.

[B105] Dotter web site. http://bioinfo.hku.hk/doc/dotter.html.

[B106] Altschul SF, Madden TL, Schaffer AA, Zhang J, Zhang Z, Miller W, Lipman DJ (1997). Gapped BLAST and PSI-BLAST: a new generation of protein database search programs. Nucleic Acids Res.

[B107] Rice P, Longden I, Bleasby A (2000). EMBOSS: the European Molecular Biology Open Software Suite. Trends Genet.

[B108] Altschul S, Gish W, Miller W, Myers E, Lipman D (1990). Basic local alignment search tool. J Mol Biol.

[B109] The Floral Genome Project – PlantTribes. http://fgpdev.huck.psu.edu/tribe.pl.

[B110] The TIGR plant gene indices web site. http://www.tigr.org/tdb/tgi/plant.shtml.

[B111] Edgar RC (2004). MUSCLE: multiple sequence alignment with high accuracy and high throughput. Nucleic Acids Res.

[B112] Swofford DL (2003). PAUP*. Phylogenetic Analysis Using Parsimony (*and Other Methods). Version 4. Sinauer Associates, Sunderland, Massachusetts.

[B113] The Global Musa Genomics Consortium. http://www.musagenomics.org.

[B114] Pertea G, Huang X, Liang F, Antonescu V, Sultana R, Karamycheva S, Lee Y, White J, Cheung F, Parvizi B (2003). TIGR Gene Indices clustering tools (TGICL): a software system for fast clustering of large EST datasets. Bioinformatics.

[B115] Birney E, Thompson JD, Gibson TJ (1996). PairWise and SearchWise: finding the optimal alignment in a simultaneous comparison of a protein profile against all DNA translation frames. Nucleic Acids Res.

[B116] Yang Z (1999). Phylogenetic analysis by maximum likelihood (PAML), version 2. University College London, England.

[B117] Goldman NaZY (1994). A codon-based model of nucleotide substitution for protein-coding DNA sequences. Mol Biol Evol.

[B118] Chase MW (2004). Monocot relationships: an overview. American Journal of Botany.

[B119] The Expressed Sequence Tags Database. http://www.ncbi.nlm.nih.gov/dbEST/.

[B120] Yuan Q, Ouyang S, Wang A, Zhu W, Maiti R, Lin H, Hamilton J, Haas B, Sultana R, Cheung F (2005). The institute for genomic research Osa1 rice genome annotation database. Plant Physiol.

