# Supplementary material for: Insights into the Musa genome: Syntenic relationships to rice and between Musa species
Source: BMC Genomics. 2008 Jan 30;9:58. doi: 10.1186/1471-2164-9-58 (PMC2270835; doi:10.1186/1471-2164-9-58)
Supplement: Additional file 1 — Supplementary Table 1. Additional list of probes used to identify the Musa BAC clones. Estimated copy numbers of these sequences in rice, Sorghum and Musa are indicated for SbRPG (Sorghum bicolor) sequences. [file 1471-2164-9-58-S1.doc]

**Supplementary Table 1.**

| **Probe and AC number** | **Putative function** | **Estimated copy number in rice by Blast analysis (Rice genes locus identifier)** | **Estimated copy number in *Sorghum* by Southern blot analysis** | **Estimated copy number in *Musa* by Southern blot analysis** | **Number of identified *Musa* BAC clones** | **Number of *Musa* BAC fingerprint groups** |
| --- | --- | --- | --- | --- | --- | --- |
|
|
| SbRPG273  DQ185898 | protease inhibitor/seed storage/LTP family | **2** Os05g06780.1 Os04g33920.1 | 1 | 2 | 6 | 2 |
|
| SbRPG663  DQ185897 | ribosomal protein | **3**  Os03g59310.1 Os07g10660.1 Os07g23800.1 | 3 | more than 2 | 32 | 5 |
|
|
| SbRPG825  DQ185899 | chlorophyll A-B binding protein type II | **6**  Os03g39610.1 Os09g17740.1 Os01g41710.1 Os01g52240.1 Os07g37550.1 Os11g13890.2 | 6 | more than 3 | 60 | 6 |
