# Supplementary material for: Insights into the Musa genome: Syntenic relationships to rice and between Musa species
Source: BMC Genomics. 2008 Jan 30;9:58. doi: 10.1186/1471-2164-9-58 (PMC2270835; doi:10.1186/1471-2164-9-58)
Supplement: Additional file 2 — Supplementary Table 2. Additional BAC clones analyzed to define Musa gene features and syntenic relationships with rice. [file 1471-2164-9-58-S2.doc]

**Supplementary Table 2.**

| **BAC number** | **Species** | **Size (in bp)** | **AC GenBank** | **Source** |
| --- | --- | --- | --- | --- |
| **MBP_71C19** | *Musa balbisiana* | 135709 | AP009325 | Gayral et al., manuscript submitted |
| **MBP_94I16** | *Musa balbisiana* | 121630 | AP009326 | Gayral et al., manuscript submitted |
| **MuG9** | *Musa acuminata* | 73268 | AY484589 | [43] |
| **MuH9** | *Musa acuminata* | 82723 | AY484588 | [43] |
