# Supplementary material for: Insights into the Musa genome: Syntenic relationships to rice and between Musa species
Source: BMC Genomics. 2008 Jan 30;9:58. doi: 10.1186/1471-2164-9-58 (PMC2270835; doi:10.1186/1471-2164-9-58)
Supplement: Additional file 3 — Supplementary Table 3. Statistics of the 17 Musa BAC clones analyzed in the present study. [file 1471-2164-9-58-S3.doc]

**Supplementary Table 3**.

| **BAC** | | **No of Coding Genes:** | | | **Other genes** | |
| --- | --- | --- | --- | --- | --- | --- |
| **BAC name** | **BAC Size (bp)** | **Total** | **Genes with match in public DB** | **Hypothetical genes*** | **Identifiable pseudogenes** | **TE** |
| **MA4_25J11** | 105,019 | 24 | 14 | 10 | 0 | 2 |
| **MA4_54N07** | 96,443 | 19 | 9 | 10 | 2 | 3 |
| **MA4_64C22** | 80,932 | 15 | 7 | 8 | 0 | 3 |
| **MA4_82I11** | 102,232 | 18 | 8 | 10 | 1 | 0 |
| **MA4_54B05** | 54,106 | 8 | 4 | 4 | 0 | 4 |
| **MBP_81C12** | 142,973 | 25 | 14 | 11 | 1 | 1 |
| **MBP_91N22** | 154,246 | 35 | 22 | 13 | 1 | 5 |
| **MA4_112I10** | 102,441 | 25 | 11 | 14 | 0 | 6 |
| **MBP_71C19** | 133,047 | 16 | 11 | 5 | 0 | 4 |
| **MBP_94I16** | 119,244 | 16 | 11 | 5 | 0 | 5 |
| **MuG9** | 73,268 | 12 | 3 | 9 | 0 | 5 |
| **MuH9** | 82,723 | 19 | 12 | 7 | 0 | 0 |
| **MA4_106O17** | 140,975 | 31 | 16 | 15 | 0 | 0 |
| **MA4_8L21** | 113,519 | 25 | 15 | 10 | 0 | 2 |
| **MA4_42M13** | 29,567 | 7 | 3 | 4 | 0 | 0 |
| **MA4_78I12** | 148,020 | 7 | 1 | 6 | 0 | 32 |
| **MA4_111B14** | 143,941 | 22 | 7 | 15 | 0 | 5 |
| **TOTAL** | 1,822,696 | 443 | 167 | 156 | 5 | 77 |

*Notes: hypothetical genes less than 100 aa and regions with similarity to hypothetical genes from other species but lacking a good open reading frame (conserved hypothetical pseudogene) are not included in the table. TE: transposable elements not calculated in the total number of coding genes.
