# Supplementary material for: Insights into the Musa genome: Syntenic relationships to rice and between Musa species
Source: BMC Genomics. 2008 Jan 30;9:58. doi: 10.1186/1471-2164-9-58 (PMC2270835; doi:10.1186/1471-2164-9-58)
Supplement: Additional file 4 — Supplementary Table 4. Annotation overview of the Musa genes. The column «Pseudogene» indicates by [1] if the gene is a pseudogene and [0] if not. Closest sequence homolog is the first similar protein sequence found by BLASTP after the sequence itself. [file 1471-2164-9-58-S4.doc]

**Supplementary Table 4.**

| **Locus name** | **Pseudogene** | **Start** | **End** | **Putative function** | **Closest sequence homolog (isolated by Blastp)** | **Blastp score** | **e-value** |
| --- | --- | --- | --- | --- | --- | --- | --- |
| F94I16.2 | [1] | 1101 | 5182 | transposon-related (monkey) | gb|AAD13304.1| polyprotein [Lycopersicon esculentum] | 222 | 8e-56 |
| F94I16.4 | [1] | 8116 | 8358 | hypothetical protein | - | - | - |
| F94I16.6 | [0] | 8798 | 11049 | phenylalanine ammonia-lyase, putative | gb|ABM67591.1| phenylalanine ammonia-lyase [Vitis vinifera] | 1124 | 0.0 |
| F94I16.8 | [0] | 11349 | 12202 | TCP family transcription factor | emb|CAE45599.1| TCP-domain protein [Antirrhinum majus subsp. majus] | 43 | 0.026 |
| F94I16.10 | [0] | 13159 | 20577 | zonadhesin-related | dbj|BAD67948.1| unknown protein [Oryza sativa (japonica cultivar-group)] | 101 | 6e-19 |
| F94I16.12 | [0] | 29067 | 31706 | chromodomain-helicase-DNA-binding family protein | gb|EAZ21463.1| hypothetical protein OsJ_004946 [Oryza sativa (japonica cultivar-group)] | 119 | 5e-26 |
| F94I16.14 | [1] | 33207 | 38169 | transposon-related (Banana streak virus) | ref|YP_233107.1| polyprotein [Banana streak GF virus] | 1922 | 0.0 |
| F94I16.16 | [0] | 39268 | 42114 | transposon-related (Banana streak virus) | ref|YP_233107.1| polyprotein [Banana streak GF virus] | 890 | 0.0 |
| F94I16.18 | [1] | 42767 | 49139 | transposon-related (Banana streak virus) | ref|YP_233107.1| polyprotein [Banana streak GF virus] | 1856 | 0.0 |
| F94I16.20 | [1] | 49441 | 50799 | transposon-related (Ty3-Gypsy) | emb|CAN76208.1| hypothetical protein [Vitis vinifera]. | 266 | 3e-69 |
| F94I16.22 | [0] | 54209 | 56602 | transcriptional regulator (MOM1-related) | gb|EAY84163.1| hypothetical protein OsI_005396 [Oryza sativa (indica cultivar-group)]. | 259 | 2e-67 |
| F94I16.24 | [1] | 57011 | 59852 | pseudogene, hypothetical protein | emb|CAN64698.1| hypothetical protein [Vitis vinifera] | 228 | 2e-57 |
| F94I16.26 | [0] | 63069 | 66507 | hypothetical protein | gb|EAZ21463.1| hypothetical protein OsJ_004946 [Oryza sativa (japonica cultivar-group)] | 127 | 1e-27 |
| F94I16.28 | [0] | 68017 | 72774 | hypothetical protein | emb|CAN64222.1| hypothetical protein [Vitis vinifera] | 60 | 7e-07 |
| F94I16.30 | [1] | 73806 | 74139 | hypothetical protein | - | - | - |
| F94I16.32 | [0] | 78063 | 78665 | auxin-responsive SAUR family protein | emb|CAE05452.3| OSJNBa0073E02.12 [Oryza sativa (japonica cultivar-group)] | 80 | 9e-14 |
| F94I16.34 | [0] | 80491 | 83257 | actin-depolymerizing factor, putative | gb|AAF60173.1|AF236068_1 actin depolymerizing factor [Elaeis guineensis] | 224 | 2e-57 |
| F94I16.36 | [1] | 84064 | 84490 | hypothetical protein | - | - | - |
| F94I16.38 | [0] | 92254 | 93778 | myb-related transcription factor, putative | gb|AAB41101.1| transcription factor Myb1 [Nicotiana tabacum] | 256 | 2e-66 |
| F94I16.40 | [0] | 97501 | 100128 | protein kinase family protein | dbj|BAD07845.1| putative wall-associated kinase [Oryza sativa (japonica cultivar-group)] | 684 | 0.0 |
| F94I16.42 | [0] | 102598 | 103133 | hypothetical protein | - | - | - |
| F94I16.44 | [0] | 105488 | 108091 | protein kinase family protein | dbj|BAD07845.1| putative wall-associated kinase [Oryza sativa (japonica cultivar-group)] | 731 | 0.0 |
| F94I16.46 | [0] | 109438 | 110678 | protein kinase family protein | gb|EAY78033.1| hypothetical protein OsI_031992 [Oryza sativa (indica cultivar-group)] | 85 | 6e-15 |
| F94I16.48 | [0] | 113308 | 115900 | protein kinase family protein | dbj|BAD07845.1| putative wall-associated kinase [Oryza sativa (japonica cultivar-group)] | 700 | 0.0 |
| C4BAM-G9.2 | [0] | 500 | 2249 | hypothetical protein, 3' partial | ref|NP_001044917.1| Os01g0867900 [Oryza sativa (japonica cultivar-group)] | 189 | 3e-47 |
| C4BAM-G9.4 | [0] | 4451 | 6608 | hypothetical protein | gb|AAK58088.1|AF380155_1 c-Fos [Ctenopharyngodon idella] | 43 | 0.018 |
| C4BAM-G9.6 | [0] | 9499 | 14693 | ribonucleoside-diphosphate reductase (ribonucleotide reductase), putative | gb|AAR95994.1| putative ribonucleotide reductase large subunit [Musa acuminata] | 1588 | 0.0 |
| C4BAM-G9.8 | [0] | 17310 | 20747 | protein kinase family protein | gb|AAR95995.1| hypothetical protein kinase [Musa acuminata] | 569 | e-160 |
| C4BAM-G9.10 | [0] | 23935 | 25748 | hypothetical protein | gb|AAR95996.1| hypothetical protein kinase [Musa acuminata] | 355 | 3e-96 |
| C4BAM-G9.12 | [1] | 28993 | 29330 | hypothetical protein | - | - | - |
| C4BAM-G9.14 | [0] | 31582 | 36891 | protein kinase family protein | gb|AAR95997.1| hypothetical protein kinase [Musa acuminata] | 751 | 0.0 |
| C4BAM-G9.16 | [0] | 37949 | 39056 | hypothetical protein | gb|AAR95998.1| hypothetical protein kinase [Musa acuminata] | 419 | e-115 |
| C4BAM-G9.18 | [0] | 39569 | 40541 | hypothetical protein | - | - | - |
| C4BAM-G9.20 | [0] | 41590 | 42016 | hypothetical protein | - | - | - |
| C4BAM-G9.22 | [0] | 42530 | 46281 | RNA recognition motif (RRM)-containing protein | gb|AAR95999.1| hypothetical protein [Musa acuminata] | 309 | 2e-82 |
| C4BAM-G9.24 | [1] | 47953 | 48242 | hypothetical protein | - | - | - |
| C4BAM-G9.26 | [0] | 48669 | 52428 | hypothetical protein | - | - | - |
| C4BAM-G9.28 | [0] | 52579 | 53526 | hypothetical protein | dbj|BAD15689.1| hypothetical protein [Oryza sativa (japonica cultivar-group)] | 36 | 0.78 |
| C4BAM-G9.30 | [1] | 53920 | 60240 | transposon-related (Ty1-Copia) | dbj|BAA22288.1| polyprotein [Oryza australiensis] | 759 | 0.0 |
| C4BAM-G9.32 | [1] | 60704 | 61720 | transposon-related (monkey) | - | - | - |
| C4BAM-G9.34 | [1] | 63976 | 66561 | transposon-related (Ty1-Copia) | gb|AAR96002.1| retrotransposon-like protein [Musa acuminata] | 569 | e-160 |
| C4BAM-G9.36 | [1] | 67467 | 67990 | transposon-related (monkey) | - | - | - |
| C4BAM-G9.38 | [1] | 69206 | 73192 | transposon-related (Ty1-Copia) | gb|ABD32582.1| Integrase, catalytic region; Zinc finger, CCHC-type; Peptidase aspartic, catalytic [Medicago truncatula] | 980 | 0.0 |
| C4BAM-H9.2 | [0] | 2 | 1929 | malate synthase, glyoxysomal (MS), 3' partial | gb|AAK07428.2|AF321286_1 malate synthase [Musa acuminata] | 999 | 0.0 |
| C4BAM-H9.4 | [0] | 4765 | 5238 | zinc finger (AN1-like) family protein | gb|AAR96005.1| hypothetical protein [Musa acuminata] | 265 | 1e-69 |
| C4BAM-H9.6 | [0] | 8233 | 14860 | phosphoglycolate phosphatase, putative | ref|NP_001053167.1| Os04g0490800 [Oryza sativa (japonica cultivar-group)] | 533 | e-149 |
| C4BAM-H9.8 | [0] | 16789 | 18915 | hAT dimerisation domain-containing protein | gb|AAR96007.1| transposase-like protein [Musa acuminata] | 1297 | 0.0 |
| C4BAM-H9.10 | [1] | 20614 | 20868 | hypothetical protein | - | - | - |
| C4BAM-H9.12 | [0] | 22864 | 23766 | ARIADNE-like protein (ubiquitin-conjugating enzyme)-related | gb|AAR96008.1| ARIADNE-like protein [Musa acuminata] | 135 | 6e-31 |
| C4BAM-H9.14 | [0] | 25343 | 26062 | hypothetical protein | - | - | - |
| C4BAM-H9.16 | [0] | 27017 | 32851 | ARIADNE-like protein (ubiquitin-conjugating enzyme), putative | emb|CAE02043.2| OJ990528_30.1 [Oryza sativa (japonica cultivar-group)] | 726 | 0.0 |
| C4BAM-H9.18 | [0] | 33811 | 35126 | hypothetical protein | ref|NP_213247.1| hypothetical protein aq_362 [Aquifex aeolicus VF5] | 38 | 0.58 |
| C4BAM-H9.20 | [0] | 36705 | 37305 | hypothetical protein | - | - | - |
| C4BAM-H9.22 | [0] | 38972 | 41656 | protein kinase family protein | gb|AAR96009.1| crinkly4-like protein [Musa acuminata] | 1757 | 0.0 |
| C4BAM-H9.24 | [0] | 41928 | 42251 | hypothetical protein | ref|XP_459918.1| hypothetical protein DEHA0E14641g [Debaryomyces hansenii CBS767] | 38 | 0.21 |
| C4BAM-H9.26 | [1] | 43379 | 43646 | hypothetical protein | - | - | - |
| C4BAM-H9.28 | [0] | 46077 | 46529 | calcium-binding protein, putative | gb|AAR96010.1| calmodulin-like protein [Musa acuminata] | 250 | 2e-65 |
| C4BAM-H9.30 | [0] | 47752 | 49525 | UbiA prenyltransferase family protein | gb|AAR96011.1| hypothetical protein [Musa acuminata] | 623 | e-177 |
| C4BAM-H9.32 | [0] | 53693 | 57587 | plant regulator RWP-RK domain-containing protein / octicosapeptide/Phox/Bem1p (PB1) domain-containing protein / nodule inception protein-related | gb|AAR96011.1| hypothetical protein [Musa acuminata] | 1844 | 0.0 |
| C4BAM-H9.34 | [0] | 59398 | 60260 | hypothetical protein | gb|AAR96012.1| hypothetical protein [Musa acuminata] | 270 | 7e-71 |
| C4BAM-H9.36 | [0] | 63294 | 64420 | F-box protein-related | gb|AAR96013.1| F-box-like protein [Musa acuminata] | 602 | e-170 |
| C4BAM-H9.38 | [1] | 68388 | 68675 | hypothetical protein | gb|ABA99375.1| expressed protein [Oryza sativa (japonica cultivar-group)] | 77 | 3e-13 |
| C4BAM-H9.40 | [0] | 69759 | 71242 | hypothetical protein | - | - | - |
| C4BAM-H9.42 | [0] | 71991 | 73442 | oxidoreductase, short-chain dehydrogenase/reductase (SDR) family protein | gb|AAR96014.1| putative short-chain hydrogenase/reductase [Musa acuminata] | 323 | 2e-86 |
| C4BAM-H9.44 | [1] | 73784 | 74029 | hypothetical protein | - | - | - |
| C4BAM-H9.46 | [0] | 77709 | 81567 | RNA recognition motif (RRM)-containing protein | gb|AAR96015.1| putative RNA-binding protein [Musa acuminata] | 511 | e-143 |
| MA4_106O17.2 | [0] | 3 | 7153 | soluble glycogen [starch] synthase, chloroplast, putative, 5' partial | emb|CAA64173.1| soluble-starch-synthase [Solanum tuberosum] | 424 | e-117 |
| MA4_106O17.4 | [0] | 8765 | 13066 | glycosyl transferase family 31 protein | emb|CAN63417.1| hypothetical protein [Vitis vinifera] | 466 | e-129 |
| MA4_106O17.6 | [0] | 15292 | 18876 | aminomethyltransferase, mitochondrial (glycine cleavage system T protein), putative | sp|P54260|GCST_SOLTU Aminomethyltransferase, mitochondrial precursor (Glycine cleavage system T protein) (GCVT) | 640 | 0.0 |
| MA4_106O17.8 | [0] | 19481 | 20943 | hypothetical protein | - | - | - |
| MA4_106O17.10 | [0] | 22906 | 26614 | amine oxidase family protein | ref|NP_001053923.1| Os04g0623300 [Oryza sativa (japonica cultivar-group)] | 720 | 0.0 |
| MA4_106O17.12 | [0] | 28387 | 29074 | hypothetical protein | ref|NP_564352.1| AGL79 (AGAMOUS-LIKE 79) [Arabidopsis thaliana] | 46 | 6e-04 |
| MA4_106O17.14 | [0] | 29398 | 29742 | plant lipid transfer/seed storage/trypsin-alpha amylase inhibitor (LTP) family protein | gb|EAZ05489.1| hypothetical protein OsI_026721 [Oryza sativa (indica cultivar-group)] | 113 | 4e-24 |
| MA4_106O17.16 | [1] | 29950 | 30321 | hypothetical protein | - | - | - |
| MA4_106O17.18 | [1] | 31792 | 32196 | hypothetical protein | - | - | - |
| MA4_106O17.20 | [0] | 32766 | 33619 | hypothetical protein | - | - | - |
| MA4_106O17.22 | [0] | 34162 | 36718 | hypothetical protein | - | - | - |
| MA4_106O17.24 | [0] | 38511 | 43138 | transcriptional factor B3 family protein | ref|NP_001058360.1| Os06g0677800 [Oryza sativa (japonica cultivar-group)] | 1119 | 0.0 |
| MA4_106O17.26 | [0] | 45326 | 51301 | hypothetical protein | ref|NP_001043426.1| Os01g0585600 [Oryza sativa (japonica cultivar-group)] | 498 | e-138 |
| MA4_106O17.28 | [0] | 54143 | 56116 | hypothetical protein | ref|NP_174332.1| phototropic-responsive NPH3 family protein [Arabidopsis thaliana] | 45 | 0.005 |
| MA4_106O17.30 | [0] | 58459 | 61100 | nonphototropic hypocotyl 3 (NPH3) family protein | emb|CAN72130.1| hypothetical protein [Vitis vinifera] | 849 | 0.0 |
| MA4_106O17.32 | [0] | 63537 | 66200 | zinc finger (C3HC4 type RING finger) family protein | dbj|BAD45521.1| zinc finger (C3HC4-type RING finger) protein-like [Oryza sativa (japonica cultivar-group)] | 347 | 6e-94 |
| MA4_106O17.34 | [0] | 69973 | 73184 | male sterility protein-related | gbEAZ41409.1| hypothetical protein OsJ_024892 [Oryza sativa (japonica cultivar-group)] | 273 | 4e-71 |
| MA4_106O17.36 | [0] | 79794 | 85831 | hypothetical protein | emb|CAN83535.1| hypothetical protein [Vitis vinifera] | 174 | 4e-41 |
| MA4_106O17.38 | [0] | 86976 | 89185 | no apical meristem (NAM) protein, putative | gb|EAZ02065.1| hypothetical protein OsI_023297 [Oryza sativa (indica cultivar-group)] | 310 | 7e-83 |
| MA4_106O17.40 | [0] | 89543 | 89963 | hypothetical protein | - | - | - |
| MA4_106O17.42 | [1] | 90129 | 90530 | hypothetical protein | - | - | - |
| MA4_106O17.44 | [1] | 93266 | 94010 | hypothetical protein | - | - | - |
| MA4_106O17.46 | [0] | 95379 | 98567 | heat shock protein DnaJ N-terminal domain-containing protein | ref|NP_001057790.1| Os06g0535300 [Oryza sativa (japonica cultivar-group)] | 656 | 0.0 |
| MA4_106O17.48 | [0] | 101443 | 102803 | hypothetical protein | ref|YP_980965.1| beta-lactamase domain protein [Polaromonas naphthalenivorans CJ2] | 38 | 0.50 |
| MA4_106O17.50 | [0] | 103851 | 104534 | hypothetical protein | ref|NP_001043031.1| Os01g0363300 [Oryza sativa (japonica cultivar-group)] | 97 | 1e-18 |
| MA4_106O17.52 | [0] | 106564 | 107928 | hypothetical protein | gb|EAZ03696.1| hypothetical protein OsI_024928 [Oryza sativa (indica cultivar-group)] | 91 | 1e-16 |
| MA4_106O17.54 | [0] | 109093 | 112288 | double-stranded RNA-binding (DsRBD) domain-containing protein | ref|NP_001054665.1| Os05g0150400 [Oryza sativa (japonica cultivar-group)] | 335 | 1e-89 |
| MA4_106O17.56 | [0] | 112852 | 114364 | hypothetical protein | - | - | - |
| MA4_106O17.58 | [0] | 114779 | 115090 | glutaredoxin family protein | ref|NP_001043047.1| Os01g0368900 [Oryza sativa (japonica cultivar-group)] | 172 | 4e-42 |
| MA4_106O17.60 | [0] | 117096 | 117407 | glutaredoxin family protein | sp|Q0IMV4|GRC14_ORYSJ Putative glutaredoxin-C14 precursor | 135 | 9e-31 |
| MA4_106O17.62 | [0] | 118474 | 119519 | hypothetical protein | gb|EAT88202.1| hypothetical protein SNOG_04442 [Phaeosphaeria nodorum SN15] | 38 | 0.71 |
| MA4_106O17.64 | [0] | 123250 | 125297 | C2 domain-containing protein / XYPPX domain-containing protein | ref|NP_001045308.1| Os01g0934100 [Oryza sativa (japonica cultivar-group)] | 160 | 3e-37 |
| MA4_106O17.66 | [0] | 126159 | 134538 | gamma-aminobutyrate transaminase, putative | gb|EAZ05978.1| hypothetical protein OsI_027210 [Oryza sativa (indica cultivar-group)] | 637 | e-180 |
| MA4_106O17.68 | [0] | 136591 | 140129 | phosphoglycerate kinase, putative | emb|CAN81988.1| hypothetical protein [Vitis vinifera] | 640 | 0.0 |
| MA4_106O17.70 | [0] | 140169 | 140969 | hypothetical protein | - | - | - |
| MA4_8L21.2 | [0] | 780 | 1578 | hypothetical protein | - | - | - |
| MA4_8L21.4 | [0] | 3088 | 10647 | zinc finger (ZPR1 type) family protein | ref|NP_001062017.1| Os08g0471900 [Oryza sativa (japonica cultivar-group)] | 667 | 0.0 |
| MA4_8L21.6 | [0] | 11591 | 14126 | ankyrin repeat family protein / methyltransferase-related | ref|NP_001051351.1| Os03g0761200 [Oryza sativa (japonica cultivar-group)] | 468 | e-130 |
| MA4_8L21.8 | [0] | 15420 | 17693 | subtilisin-like serine proteinase, putative | ref|NP_001051353.1| Os03g0761500 [Oryza sativa (japonica cultivar-group)] | 1018 | 0.0 |
| MA4_8L21.10 | [0] | 17817 | 19011 | hypothetical protein | - | - | - |
| MA4_8L21.12 | [0] | 19611 | 24004 | transport protein-related | gb|AAT78760.1| putative transport protein [Oryza sativa (japonica cultivar-group)] | 366 | 5e-99 |
| MA4_8L21.14 | [1] | 25124 | 25510 | hypothetical protein | ref|NP_001058751.1| Os07g0114300 [Oryza sativa (japonica cultivar-group)] | 74 | 3e-12 |
| MA4_8L21.16 | [1] | 27772 | 28200 | hypothetical protein | emb|CAN62217.1| hypothetical protein [Vitis vinifera] | 75 | 2e-12 |
| MA4_8L21.18 | [1] | 29065 | 32181 | transposon-related (Ty1-Copia) | emb|CAN74718.1| hypothetical protein [Vitis vinifera] | 141 | 5e-32 |
| MA4_8L21.20 | [0] | 35761 | 36825 | hypothetical protein | emb|CAN72960.1| hypothetical protein [Vitis vinifera] | 267 | 1e-69 |
| MA4_8L21.22 | [0] | 38923 | 45024 | hypothetical protein | ref|NP_001051363.1| Os03g0762900 [Oryza sativa (japonica cultivar-group)] | 332 | 8e-89 |
| MA4_8L21.24 | [0] | 46968 | 54864 | casein kinase II alpha subunit (protein kinase), putative | emb|CAN71837.1| hypothetical protein [Vitis vinifera] | 561 | e-158 |
| MA4_8L21.26 | [0] | 57311 | 58572 | basic helix-loop-helix (bHLH) family protein | gb|ABA99756.1| Helix-loop-helix DNA-binding domain containing protein [Oryza sativa (japonica cultivar-group)] | 153 | 2e-35 |
| MA4_8L21.28 | [0] | 58880 | 59374 | hypothetical protein | ref|NP_492186.3| Non-muscle MYosin family member (nmy-2) [Caenorhabditis elegans] | 37 | 0.42 |
| MA4_8L21.30 | [0] | 59811 | 65518 | hypothetical protein | emb|CAN75759.1| hypothetical protein [Vitis vinifera] | 839 | 0.0 |
| MA4_8L21.32 | [0] | 67504 | 69240 | myb DNA-binding domain-containing protein | emb|CAN81424.1| hypothetical protein [Vitis vinifera] | 243 | 2e-62 |
| MA4_8L21.34 | [0] | 77296 | 78041 | hypothetical protein | ref|YP_464490.1| excinuclease ABC, C subunit [Anaeromyxobacter dehalogenans 2CP-C] | 36 | 0.78 |
| MA4_8L21.36 | [0] | 78796 | 79848 | zinc finger (Dof type) family protein | emb|CAN72540.1| hypothetical protein [Vitis vinifera] | 213 | 3e-53 |
| MA4_8L21.38 | [0] | 81410 | 82827 | hypothetical protein | ref|NP_001051384.1| Os03g0766800 [Oryza sativa (japonica cultivar-group)] | 48 | 4e-04 |
| MA4_8L21.40 | [0] | 85578 | 87065 | WD-40 repeat family protein | emb|CAN61238.1| hypothetical protein [Vitis vinifera] | 469 | e-130 |
| MA4_8L21.42 | [0] | 88756 | 92200 | cell cycle-regulated protein-related | emb|CAN60895.1| hypothetical protein [Vitis vinifera] | 288 | 3e-76 |
| MA4_8L21.44 | [0] | 93630 | 94628 | thioredoxin family protein | ref|NP_001051387.1| Os03g0767500 [Oryza sativa (japonica cultivar-group)] | 244 | 1e-63 |
| MA4_8L21.46 | [0] | 95379 | 96826 | hypothetical protein | gb|EAZ28704.1| hypothetical protein OsJ_012187 [Oryza sativa (japonica cultivar-group)] | 79 | 2e-13 |
| MA4_8L21.48 | [0] | 99434 | 103133 | cold acclimation protein-related | ref|NP_001051390.1| Os03g0767800 [Oryza sativa (japonica cultivar-group)] | 128 | 1e-28 |
| MA4_8L21.50 | [0] | 104552 | 105817 | hypothetical protein | gb|EAZ24887.1| hypothetical protein OsJ_008370 [Oryza sativa (japonica cultivar-group)] | 47 | 5e-04 |
| MA4_8L21.52 | [0] | 106536 | 109569 | hypothetical protein | - | - | - |
| MA4_8L21.54 | [0] | 110140 | 112001 | transporter-related | ref|NP_001050703.1| Os03g0626700 [Oryza sativa (japonica cultivar-group)] | 205 | 3e-51 |
| MA4_8L21.56 | [0] | 112947 | 113346 | phytosulfokine family protein | ref|NP_001050886.1| Os03g0675600 [Oryza sativa (japonica cultivar-group)] >gi|18202914|sp|Q9FRF9|PSK3_ORYSJ Phytosulfokines 3 precursor | 57 | 3e-07 |
| MA4_42M13.2 | [0] | 622 | 2233 | peptidylprolyl isomerase, FKBP-type family protein | ref|NP_001046016.1| Os02g0168700 [Oryza sativa (japonica cultivar-group)] | 285 | 2e-75 |
| MA4_42M13.4 | [0] | 4068 | 5731 | hypothetical protein | ref|NP_523475.3| Salivary gland secretion 1 CG3047-PA [Drosophila melanogaster] | 39 | 0.61 |
| MA4_42M13.6 | [0] | 7093 | 10443 | porphobilinogen deaminase, chloroplast (hydroxymethylbilane synthase) (pre-uroporphyrinogen synthase), putative | gb|EAZ21887.1| hypothetical protein OsJ_005370 [Oryza sativa (japonica cultivar-group)] | 531 | e-149 |
| MA4_42M13.8 | [0] | 11167 | 13761 | porphobilinogen deaminase, chloroplast (hydroxymethylbilane synthase) (pre-uroporphyrinogen synthase), putative | gb|EAZ21887.1| hypothetical protein OsJ_005370 [Oryza sativa (japonica cultivar-group)] | 473 | e-132 |
| MA4_42M13.10 | [0] | 13928 | 16005 | hypothetical protein | ref|NP_001046018.1| Os02g0168900 [Oryza sativa (japonica cultivar-group)] | 197 | 3e-49 |
| MA4_42M13.12 | [0] | 17661 | 22785 | DNA helicase homolog, putative | gb|AAM92800.1| putative DNA helicase homolog [Oryza sativa (japonica cultivar-group)] | 1901 | 0.0 |
| MA4_42M13.14 | [0] | 26811 | 28847 | hypothetical protein | emb|CAJ44248.1| 3-alpha-L-fucosyltransferase [Zea mays] | 39 | 0.56 |
| MA4_78I12.2 | [1] | 579 | 3529 | transposon-related (monkey) | ref|XP_001465352.1| PIWI-like protein 1 [Leishmania infantum JPCM5] | 44 | 0.043 |
| MA4_78I12.4 | [1] | 3548 | 5301 | transposon-related (monkey) | - | - | - |
| MA4_78I12.6 | [1] | 5774 | 6952 | hypothetical protein | - | - | - |
| MA4_78I12.8 | [1] | 7493 | 9274 | transposon-related (Ty3-Gypsy) | emb|CAN68669.1| hypothetical protein [Vitis vinifera] | 357 | 2e-96 |
| MA4_78I12.10 | [0] | 11969 | 14776 | hypothetical protein | ref|XP_547945.2| PREDICTED: similar to Tyrosine-protein phosphat..phosphatase, non-receptor type 21 (Protein-tyrosine phosphatase D1) [Canis familiaris] | 42 | 0.11 |
| MA4_78I12.12 | [1] | 14968 | 20262 | transposon-related (Ty3-Gypsy) | gb|AAY99339.1| pol-polyprotein [Silene latifolia] | 1032 | 0.0 |
| MA4_78I12.14 | [1] | 20800 | 23412 | transposon-related (Ty3-Gypsy) | emb|CAN76793.1| hypothetical protein [Vitis vinifera] | 924 | 0.0 |
| MA4_78I12.16 | [0] | 24177 | 26916 | hypothetical protein | emb|CAN63411.1| hypothetical protein [Vitis vinifera] | 69 | 1e-09 |
| MA4_78I12.18 | [1] | 27113 | 31708 | transposon-related (Ty1-Copia) | gb|AAO73527.1| gag-pol polyprotein [Glycine max] | 1189 | 0.0 |
| MA4_78I12.20 | [1] | 32292 | 36739 | transposon-related (monkey) | - | - | - |
| MA4_78I12.22 | [1] | 37229 | 41323 | transposon-related (Ty3-Gypsy) | emb|CAN76793.1| hypothetical protein [Vitis vinifera] | 1128 | 0.0 |
| MA4_78I12.24 | [1] | 42865 | 45794 | transposon-related (Ty1-Copia) | emb|CAN83181.1| hypothetical protein [Vitis vinifera] | 437 | e-120 |
| MA4_78I12.26 | [1] | 47000 | 47787 | transposon-related (monkey) | - | - | - |
| MA4_78I12.28 | [1] | 48707 | 52252 | transposon-related (Ty1-Copia) | emb|CAN76317.1| hypothetical protein [Vitis vinifera] | 874 | 0.0 |
| MA4_78I12.30 | [1] | 53046 | 55400 | transposon-related (Ty3-Gypsy) | emb|CAN81876.1| hypothetical protein [Vitis vinifera] | 202 | 9e-50 |
| MA4_78I12.32 | [1] | 57387 | 58550 | transposon-related (Ty1-Copia) | emb|CAN65058.1| hypothetical protein [Vitis vinifera] | 344 | 1e-92 |
| MA4_78I12.34 | [1] | 59947 | 64048 | transposon-related (monkey) | - | - | - |
| MA4_78I12.36 | [1] | 64630 | 65328 | transposon-related (Ty1-Copia) | emb|CAN65059.1| hypothetical protein [Vitis vinifera] | 184 | 5e-45 |
| MA4_78I12.38 | [1] | 68190 | 69457 | transposon-related (monkey) | - | - | - |
| MA4_78I12.40 | [1] | 73889 | 77383 | transposon-related (monkey) | gb|AAT38724.1| Putative retrotransposon protein, identical [Solanum demissum] | 1305 | 0.0 |
| MA4_78I12.42 | [0] | 79036 | 81344 | hypothetical protein | ref|NP_983334.1| ACL070Cp [Ashbya gossypii ATCC 10895] | 36 | 0.60 |
| MA4_78I12.44 | [0] | 81485 | 81799 | hypothetical protein | ref|XP_718010.1| agglutinin-like ALS1 protein [Candida albicans SC5314] | 36 | 0.78 |
| MA4_78I12.46 | [1] | 82435 | 86682 | transposon-related (Ty1-Copia) | emb|CAN66076.1| hypothetical protein [Vitis vinifera] | 169 | 2e-39 |
| MA4_78I12.48 | [1] | 86988 | 90057 | transposon-related (monkey) | - | - | - |
| MA4_78I12.50 | [1] | 92907 | 93851 | transposon-related (Ty1-Copia) | emb|CAN74868.1| hypothetical protein [Vitis vinifera] | 205 | 4e-51 |
| MA4_78I12.52 | [1] | 94142 | 97765 | transposon-related (monkey) | - | - | - |
| MA4_78I12.54 | [1] | 100090 | 100754 | hypothetical protein | - | - | - |
| MA4_78I12.56 | [1] | 100985 | 101753 | transposon-related (monkey) | - | - | - |
| MA4_78I12.58 | [1] | 104794 | 105744 | transposon-related (Ty1-Copia) | emb|CAN74868.1| hypothetical protein [Vitis vinifera] | 222 | 3e-56 |
| MA4_78I12.60 | [1] | 106528 | 108507 | transposon-related (monkey) | - | - | - |
| MA4_78I12.62 | [1] | 108784 | 111756 | transposon-related (Ty1-Copia) | emb|CAN63777.1| hypothetical protein [Vitis vinifera] | 402 | e-110 |
| MA4_78I12.64 | [1] | 112993 | 113496 | transposon-related (monkey) | - | - | - |
| MA4_78I12.66 | [1] | 115245 | 116912 | transposon-related (Ty1-Copia) | gb|AAN40025.1| putative gag-pol polyprotein [Zea mays] | 500 | e-139 |
| MA4_78I12.68 | [0] | 117640 | 120153 | hypothetical protein | emb|CAN71614.1| hypothetical protein [Vitis vinifera] | 55 | 1e-05 |
| MA4_78I12.70 | [1] | 120652 | 125271 | transposon-related (Ty1-Copia) | gb|AAO73527.1| gag-pol polyprotein [Glycine max] | 1164 | 0.0 |
| MA4_78I12.72 | [0] | 125519 | 127083 | hypothetical protein | - | - | - |
| MA4_78I12.74 | [1] | 127852 | 132270 | transposon-related (Ty1-Copia) | emb|CAN60490.1| hypothetical protein [Vitis vinifera] | 533 | e-149 |
| MA4_78I12.76 | [1] | 134037 | 138680 | transposon-related (Ty1-Copia) | emb|CAN76317.1| hypothetical protein [Vitis vinifera] | 1283 | 0.0 |
| MA4_78I12.78 | [1] | 139737 | 140872 | transposon-related (monkey) | ref|XP_700564.2| PREDICTED: similar to photomedin-1 [Danio rerio] | 45 | 0.007 |
| MA4_78I12.80 | [0] | 142896 | 143935 | hypothetical protein | - | - | - |
| MA4_78I12.82 | [1] | 144914 | 148009 | transposon-related (Ty1-Copia) | emb|CAN60490.1| hypothetical protein [Vitis vinifera] | 739 | 0.0 |
| MA4_111B14.2 | [0] | 1949 | 2549 | protein kinase, putative | gb|EAY99838.1| hypothetical protein OsI_021071 [Oryza sativa (indica cultivar-group)] | 237 | 3e-61 |
| MA4_111B14.4 | [0] | 8651 | 9491 | hypothetical protein | - | - | - |
| MA4_111B14.6 | [0] | 10547 | 14111 | nitrogen regulation family protein | gb|EAY93387.1| hypothetical protein OsI_014620 [Oryza sativa (indica cultivar-group)] | 142 | 1e-32 |
| MA4_111B14.8 | [0] | 14994 | 16121 | nitrogen regulation family protein | ref|NP_199608.1| nitrogen regulation family protein [Arabidopsis thaliana] | 73 | 4e-12 |
| MA4_111B14.10 | [0] | 17487 | 19923 | hypothetical protein | emb|CAD39831.3| OSJNBa0079F16.4 [Oryza sativa(japonica cultivar-group)] | 275 | 2e-72 |
| MA4_111B14.12 | [0] | 20319 | 23443 | hypothetical protein | - | - | - |
| MA4_111B14.14 | [0] | 24054 | 25125 | C2 domain-containing protein / protein kinase-related | ref|NP_567956.1| C2 domain-containing protein [Arabidopsis thaliana] | 172 | 1e-41 |
| MA4_111B14.16 | [1] | 28241 | 30350 | transposon-related (monkey) | - | - | - |
| MA4_111B14.18 | [0] | 31836 | 33297 | glycosyl transferase family 43 protein | emb|CAI93190.1| glycosyltransferase [Saccharum officinarum] | 435 | e-120 |
| MA4_111B14.20 | [1] | 38202 | 38483 | hypothetical protein | - | - | - |
| MA4_111B14.22 | [0] | 41061 | 49652 | oxidoreductase, 2OG-Fe(II) oxygenase family protein | emb|CAI93190.1| glycosyltransferase [Saccharum officinarum] | 435 | e-120 |
| MA4_111B14.24 | [0] | 50395 | 53483 | hypothetical protein | ref|XP_001127437.1| PREDICTED: similar to Hornerin [Homo sapiens] | 40 | 0.45 |
| MA4_111B14.26 | [1] | 54257 | 55044 | transposon-related (monkey) | - | - | - |
| MA4_111B14.28 | [0] | 57642 | 58243 | hypothetical protein | - | - | - |
| MA4_111B14.30 | [0] | 64489 | 74648 | hypothetical protein | gb|AAF26129.1|AC011620_5 unknown protein [Arabidopsis thaliana] | 795 | 0.0 |
| MA4_111B14.32 | [0] | 80092 | 80604 | hypothetical protein | - | - | - |
| MA4_111B14.34 | [0] | 80872 | 81372 | hypothetical protein | - | - | - |
| MA4_111B14.36 | [0] | 84079 | 86763 | hypothetical protein | gb|EAZ27481.1| hypothetical protein OsJ_010964 [Oryza sativa (japonica cultivar-group)] | 404 | e-110 |
| MA4_111B14.38 | [0] | 88474 | 91752 | U-box domain-containing protein | emb|CAN69463.1| hypothetical protein [Vitis vinifera] | 205 | 2e-50 |
| MA4_111B14.40 | [1] | 91883 | 92127 | hypothetical protein | - | - | - |
| MA4_111B14.42 | [0] | 96176 | 97386 | hypothetical protein | gb|AAX49610.1| RPGR [Rattus norvegicus] | 40 | 0.28 |
| MA4_111B14.44 | [0] | 97463 | 102298 | hypothetical protein | gb|ABA94028.1| expressed protein [Oryza sativa (japonica cultivar-group)] | 87 | 2e-16 |
| MA4_111B14.46 | [0] | 103389 | 107786 | hypothetical protein | ref|NP_196843.1| NEF1 (NO EXINE FORMATION 1) [Arabidopsis thaliana] | 780 | 0.0 |
| MA4_111B14.48 | [0] | 112800 | 116103 | leucine-rich repeat-containing protein kinase family protein | emb|CAN68268.1| hypothetical protein [Vitis vinifera] | 1097 | 0.0 |
| MA4_111B14.50 | [0] | 118781 | 119830 | ubiquinol-cytochrome c reductase iron-sulfur subunit, mitochondrial (Rieske iron-sulfur protein), putative | sp|P49727|UCRI_MAIZE Ubiquinol-cytochrome c reductase iron-sulfur subunit, mitochondrial precursor (Rieske iron-sulfur protein) (RISP) | 387 | e-106 |
| MA4_111B14.52 | [0] | 121123 | 121546 | hypothetical protein | - | - | - |
| MA4_111B14.54 | [1] | 124165 | 125262 | transposon-related (Mutator) | gb|AAP53948.1| transposon protein, putative, Mutator sub-class [Oryza sativa (japonica cultivar-group)] | 154 | 2e-35 |
| MA4_111B14.56 | [0] | 129460 | 130329 | hypothetical protein | emb|CAE05150.2| OSJNBa0039C07.6 [Oryza sativa(japonica cultivar-group)] | 188 | 6e-46 |
| MA4_111B14.58 | [1] | 134765 | 135502 | pseudogene, hypothetical protein | - | - | - |
| MA4_111B14.60 | [1] | 136028 | 137306 | hypothetical protein | dbj|BAB01086.1| unnamed protein product [Arabidopsis thaliana] | 87 | 3e-16 |
| MA4_111B14.62 | [1] | 137795 | 139329 | pseudogene, NADH dehydrogenase related | - | - | - |
| MA4_111B14.64 | [0] | 141355 | 142191 | hypothetical protein | ref|NP_176901.1| unknown protein [Arabidopsis thaliana] | 295 | 2e-78 |
| MA4_25J11.2 | [0] | 2 | 2747 | AMP-dependent synthetase and ligase family protein, 5' partial | emb|CAN60763.1| hypothetical protein [Vitis vinifera] | 287 | 2e-75 |
| MA4_25J11.4 | [0] | 7126 | 14463 | FtsJ-like methyltransferase family protein | ref|NP_001056350.1| Os05g0567400 [Oryza sativa(japonica cultivar-group)] | 843 | 0.0 |
| MA4_25J11.6 | [1] | 16860 | 20015 | transposon-related (Ty1-Copia) | emb|CAN63026.1| hypothetical protein [Vitis vinifera] | 321 | 3e-85 |
| MA4_25J11.8 | [0] | 22147 | 26245 | GTPase family protein | gb|ABB45853.1| hypothetical protein [Thellungiella halophila] | 491 | e-137 |
| MA4_25J11.10 | [0] | 26558 | 27690 | hypothetical protein | - | - | - |
| MA4_25J11.12 | [0] | 27722 | 29314 | (1-4)-beta-mannan endohydrolase (mannan endo-1,4-beta-mannosidase), putative | sp|Q0JKM9|MAN1_ORYSJ Mannan endo-1,4-beta-mannosidase precursor (Beta-mannanase 1) (Endo-beta-1,4-mannanase 1) (OsMAN1) | 525 | e-147 |
| MA4_25J11.14 | [0] | 29724 | 33616 | ADP-glucose pyrophosphorylase (glucose-1-phosphate adenylyltransferase) large subunit, putative | gb|AAB91468.1| ADP-glucose pyrophosphorylase large subunit 2 [Citrullus lanatus] | 691 | 0.0 |
| MA4_25J11.16 | [0] | 33831 | 34623 | hypothetical protein | gb|AAD56042.1|AF184598_1 ADP-glucose pyrophosphorylase large subunit [Citrus unshiu] | 40 | 0.063 |
| MA4_25J11.18 | [0] | 35626 | 36423 | chlorophyll A-B binding protein (CAB), putative | gb|ABF17940.1| putative chloroplast chlorophyll a/b-binding prot... | 508 | e-142 |
| MA4_25J11.20 | [0] | 38525 | 41453 | protein kinase family protein | ref|NP_001064960.1| Os10g0497600 [Oryza sativa (japonica cultivar-group)] | 636 | e-180 |
| MA4_25J11.22 | [0] | 43957 | 45214 | paxneb-related protein | ref|NP_001058161.1| Os06g0639600 [Oryza sativa (japonica cultivar-group)] | 233 | 2e-59 |
| MA4_25J11.24 | [0] | 46106 | 49982 | paxneb-related protein | gb|EAZ37769.1| hypothetical protein OsJ_021252 [Oryza sativa (japonica cultivar-group)] | 96 | 4e-18 |
| MA4_25J11.26 | [0] | 52944 | 59317 | pre-mRNA processing protein-related | gb|EAZ42567.1| hypothetical protein OsJ_026050 [Oryza sativa (japonica cultivar-group)] | 531 | e-149 |
| MA4_25J11.28 | [0] | 60048 | 63967 | hypothetical protein | gb|EAZ06818.1| hypothetical protein OsI_028050 [Oryza sativa (indica cultivar-group)] | 67 | 7e-09 |
| MA4_25J11.30 | [1] | 64719 | 65622 | hypothetical protein | - | - | - |
| MA4_25J11.32 | [0] | 66750 | 67931 | hypothetical protein | ref|NP_198901.1| UPM1 (UROPHORPHYRIN METHYLASE 1); uroporphyrin-III C-methyltransferase [Arabidopsis thaliana] | 39 | 0.25 |
| MA4_25J11.34 | [0] | 68338 | 70131 | eukaryotic translation initiation factor 3 (eIF-3), putative | gb|AAU10658.1| putative eukaryotic translation initiation (eIF3d) [Oryza sativa (japonica cultivar-group)] | 684 | 0.0 |
| MA4_25J11.36 | [0] | 70258 | 70924 | hypothetical protein | - | - | - |
| MA4_25J11.38 | [0] | 73983 | 74958 | hypothetical protein | - | - | - |
| MA4_25J11.40 | [0] | 75411 | 76363 | hypothetical protein | ref|NP_001008750.1| type I keratin KA11 [Rattus norvegicus] | 41 | 0.062 |
| MA4_25J11.42 | [1] | 76621 | 77334 | hypothetical protein | - | - | - |
| MA4_25J11.44 | [0] | 77585 | 82562 | mitogen-activated protein kinase (MAPK), putative | gb|EAY75052.1| hypothetical protein OsI_002899 [Oryza sativa (indica cultivar-group)] | 847 | 0.0 |
| MA4_25J11.46 | [0] | 84643 | 85964 | DNA-binding WRKY domain-containing protein | emb|CAN72742.1| hypothetical protein [Vitis vinifera] | 236 | 3e-60 |
| MA4_25J11.48 | [0] | 86142 | 87886 | hypothetical protein | - | - | - |
| MA4_25J11.50 | [0] | 91461 | 92405 | 6-phosphogluconate dehydrogenase NAD-binding domain-containing protein | ref|NP_001056339.1| Os05g0565500 [Oryza sativa (japonica cultivar-group)] | 301 | 7e-80 |
| MA4_25J11.52 | [0] | 92782 | 93749 | hypothetical protein | gb|EAZ35294.1| hypothetical protein OsJ_018777 [Oryza sativa (japonica cultivar-group)] | 262 | 2e-68 |
| MA4_25J11.54 | [0] | 99043 | 100231 | vacuolar protein sorting 16 (VPS16) family protein | gb|AAD32769.1|AC007661_6 putative vacuolar protein sorting-associated protein (VPS16) [Arabidopsis thaliana] | 301 | 6e-80 |
| MA4_25J11.56 | [0] | 101248 | 104920 | vacuolar protein sorting 16 (VPS16) family protein | ref|NP_565879.1| VCL1 (VACUOLELESS 1) [Arabidopsis thaliana] | 335 | 1e-90 |
| MA4_54N07.2 | [0] | 2293 | 4206 | hypothetical protein, 5' partial | gb|EAZ11319.1| hypothetical protein OsJ_001144 [Oryza sativa (japonica cultivar-group)] | 228 | 2e-58 |
| MA4_54N07.4 | [0] | 7259 | 15794 | phosphatidylinositol transfer protein, putative | dbj|BAD07999.1| putative phosphatidylinositol/ phophatidylcholine transfer protein [Oryza sativa (japonica cultivar-group)] | 835 | 0.0 |
| MA4_54N07.6 | [0] | 17041 | 17939 | hypothetical protein | - | - | - |
| MA4_54N07.8 | [0] | 20272 | 21923 | transcriptional regulator family protein | ref|NP_001057623.1| Os06g0472400 [Oryza sativa (japonica cultiva... | 442 | e-122 |
| MA4_54N07.10 | [0] | 23411 | 25927 | hypothetical protein | gb|EAZ00916.1| hypothetical protein OsI_022148 [Oryza sativa (indica cultivar-group)] | 504 | e-141 |
| MA4_54N07.12 | [0] | 27512 | 28119 | hypothetical protein | - | - | - |
| MA4_54N07.14 | [1] | 37020 | 37528 | cytidyltransferase domain-containg protein | ref|NP_179417.1| Atcoad (4-phosphopantetheine adenylyltransferase); nucleotidyltransferase/ pantetheine-phosphate adenylyltransferase [Arabidopsis thaliana] | 120 | 2e-26 |
| MA4_54N07.16 | [0] | 38318 | 39695 | basic helix-loop-helix (bHLH) family protein | gb|EAZ24332.1| hypothetical protein OsJ_007815 [Oryza sativa (japonica cultivar-group)] | 144 | 1e-32 |
| MA4_54N07.18 | [0] | 39711 | 40818 | protein kinase-related | ref|NP_001041848.1| Os01g0117100 [Oryza sativa (japonica cultivar-group)] >gi|53791461|dbj|BAD52513.1| putative rust resistance kinase Lr10 | 67 | 2e-10 |
| MA4_54N07.20 | [1] | 40899 | 41354 | transposon-related (Ty1-Copia) | emb|CAN68578.1| hypothetical protein [Vitis vinifera] | 157 | 3e-37 |
| MA4_54N07.22 | [0] | 41761 | 43770 | hypothetical protein | gb|ABC87794.1| breast and ovarian cancer susceptibility 1 [Dinomys branickii] | 40 | 0.18 |
| MA4_54N07.24 | [0] | 43903 | 46338 | U-box domain-containing protein | ref|NP_001057649.1| Os06g0480000 [Oryza sativa (japonica cultivar-group)] | 407 | e-111 |
| MA4_54N07.26 | [0] | 53163 | 53531 | hypothetical protein | ref|XP_001236547.1| PREDICTED: hypothetical protein [Gallus gallus] | 40 | 0.054 |
| MA4_54N07.28 | [1] | 54936 | 56035 | pseudogene, beta-1,3-glucanase-related | pir||T05722 licheninase (EC 3.2.1.73) - barley | 55 | 7e-06 |
| MA4_54N07.30 | [0] | 56234 | 58455 | kinesin light chain-related | gb|ABF70051.1| kinesin light chain-related [Musa acuminata] | 1314 | 0.0 |
| MA4_54N07.32 | [0] | 61195 | 62475 | hypothetical protein | ref|NP_001047887.1| Os02g0709100 [Oryza sativa (japonica cultivar-group)] | 199 | 4e-49 |
| MA4_54N07.34 | [0] | 62892 | 65228 | exostosin family protein | gb|ABF70053.1| exostosin family protein [Musa acuminata] | 986 | 0.0 |
| MA4_54N07.36 | [0] | 66318 | 68765 | protein kinase family protein | gb|ABF70054.1| protein kinase family protein [Musa acuminata] | 810 | 0.0 |
| MA4_54N07.38 | [1] | 70200 | 71087 | transposon-related (Ty1-Copia) | - | - | - |
| MA4_54N07.40 | [0] | 73475 | 75686 | hypothetical protein | ref|NP_001057685.1| Os06g0494000 [Oryza sativa (japonica cultivar-group)] | 88 | 2e-16 |
| MA4_54N07.42 | [0] | 77098 | 86562 | paired amphipathic helix repeat-containing protein / transcription regulator-related | gb|EAZ10246.1| hypothetical protein OsJ_000071 [Oryza sativa (japonica cultivar-group)] | 1539 | 0.0 |
| MA4_54N07.44 | [0] | 90552 | 91137 | hypothetical protein | - | - | - |
| MA4_54N07.46 | [0] | 91827 | 92589 | hypothetical protein | - | - | - |
| MA4_54N07.48 | [0] | 94570 | 95277 | GTP-binding protein, putative | emb|CAN75016.1| hypothetical protein [Vitis vinifera] | 228 | 2e-58 |
| MA4_64C22.2 | [0] | 1258 | 2340 | cytochrome P450 family protein | ref|NP_001058164.1| Os06g0640500 [Oryza sativa (japonica cultivar-group)] | 195 | 5e-48 |
| MA4_64C22.4 | [1] | 9622 | 13752 | transposon-related (Ty1-Copia) | dbj|BAA11674.1| unnamed protein product [Nicotiana tabacum] | 1208 | 0.0 |
| MA4_64C22.6 | [0] | 15404 | 16534 | hypothetical protein | ref|NP_001060232.1| Os07g0606900 [Oryza sativa japonica cultivar-group)] | 66 | 8e-10 |
| MA4_64C22.8 | [0] | 18964 | 20568 | cytochrome P450 family protein | ref|NP_001058164.1| Os06g0640500 [Oryza sativa (japonica cultivar-group)] | 403 | e-110 |
| MA4_64C22.10 | [0] | 22685 | 24427 | hypothetical protein | - | - | - |
| MA4_64C22.12 | [0] | 26698 | 28302 | cytochrome P450 family protein | ref|NP_001058164.1| Os06g0640500 [Oryza sativa (japonica cultivar-group)] | 407 | e-111 |
| MA4_64C22.14 | [1] | 29893 | 34526 | transposon-related (monkey) | - | - | - |
| MA4_64C22.16 | [1] | 34814 | 35864 | transposon-related (Ty3-Gypsy) | emb|CAN74304.1| hypothetical protein [Vitis vinifera] | 157 | 1e-36 |
| MA4_64C22.18 | [0] | 36234 | 38331 | hypothetical protein | gb|AAC28207.1| T24H24.13 gene product [Arabidopsis thaliana] >gi|7267161|emb|CAB77873.1| putative transposon protein | 38 | 0.79 |
| MA4_64C22.20 | [0] | 39866 | 41174 | mitochondrial inner membrane preprotein translocase (TIM23) component-related | emb|CAN66866.1| hypothetical protein [Vitis vinifera] | 180 | 2e-44 |
| MA4_64C22.22 | [0] | 41964 | 42403 | hypothetical protein | - | - | - |
| MA4_64C22.24 | [0] | 42754 | 43763 | mitochondrial inner membrane preprotein translocase (TIM23) component-related | ref|NP_001044329.1| Os01g0762600 [Oryza sativa (japonica cultivar-group)] | 130 | 2e-29 |
| MA4_64C22.26 | [0] | 45675 | 47044 | hypothetical protein | emb|CAN70568.1| hypothetical protein [Vitis vinifera] | 107 | 3e-22 |
| MA4_64C22.28 | [0] | 50993 | 53436 | hypothetical protein | - | - | - |
| MA4_64C22.30 | [1] | 55918 | 58588 | pseudogene, hypothetical protein | gb|ABQ14917.1| Ycf2 [Rhodoleia championii] | 76 | 1e-12 |
| MA4_64C22.32 | [0] | 59772 | 60449 | hypothetical protein | emb|CAN69116.1| hypothetical protein [Vitis vinifera] | 64 | 1e-08 |
| MA4_64C22.34 | [0] | 62181 | 65079 | hypothetical protein | ref|ZP_02057454.1| hypothetical protein MchlDRAFT_4230 [Methylobacterium chloromethanicum CM4] | 40 | 0.63 |
| MA4_64C22.36 | [1] | 65706 | 67500 | pseudogene, hypothetical protein | - | - | - |
| MA4_64C22.38 | [0] | 67781 | 69067 | F-box family protein | emb|CAN69116.1| hypothetical protein [Vitis vinifera] | 124 | 2e-26 |
| MA4_64C22.40 | [0] | 75011 | 76276 | F-box family protein | dbj|BAD54292.1| hypothetical protein [Oryza sativa (japonica cultivar-group)] | 88 | 2e-15 |
| MA4_64C22.42 | [1] | 80023 | 80262 | hypothetical protein, 3' partial | ref|NP_001058751.1| Os07g0114300 [Oryza sativa (japonica cultivar-group)] | 72 | 1e-11 |
| MA4_82I11.4 | [0] | 1142 | 1955 | hypothetical protein | - | - | - |
| MA4_82I11.6 | [0] | 3778 | 5233 | hypothetical protein | - | - | - |
| MA4_82I11.10 | [0] | 5757 | 8311 | hypothetical protein | emb|CAN74035.1| hypothetical protein [Vitis vinifera] | 152 | 2e-35 |
| MA4_82I11.12 | [0] | 10492 | 11396 | hypothetical protein | - | - | - |
| MA4_82I11.16 | [0] | 12720 | 14834 | GDSL-motif lipase/hydrolase family protein | gb|EAY75691.1| hypothetical protein OsI_003538 [Oryza sativa (indica cultivar-group)] | 504 | e-141 |
| MA4_82I11.18 | [0] | 16917 | 26713 | protein kinase family protein | ref|NP_567074.2| ankyrin protein kinase, putative [Arabidopsis t... | 565 | e-159 |
| MA4_82I11.20 | [0] | 27049 | 28538 | hypothetical protein | - | - | - |
| MA4_82I11.22 | [0] | 29003 | 33768 | hypothetical protein | ref|NP_001044243.1| Os01g0748900 [Oryza sativa (japonica cultivar-group)] | 668 | 0.0 |
| MA4_82I11.24 | [0] | 34962 | 37499 | hypothetical protein | ref|NP_564333.1| CAD1 (CONSTITUTIVELY ACTIVATED CELL DEATH 1); o... | 81 | 2e-13 |
| MA4_82I11.26 | [0] | 38082 | 45109 | protein kinase family protein | emb|CAN66180.1| hypothetical protein [Vitis vinifera] | 629 | e-178 |
| MA4_82I11.28 | [0] | 45900 | 48223 | hypothetical protein | - | - | - |
| MA4_82I11.30 | [0] | 48624 | 49850 | hypothetical protein | ref|XP_001276523.1| C2H2 zinc finger protein [Aspergillus clavatus NRRL 1] | 37 | 0.79 |
| MA4_82I11.32 | [1] | 50782 | 51051 | hypothetical protein | - | - | - |
| MA4_82I11.36 | [0] | 61904 | 63306 | protein kinase-related | gb|AAU10526.1| putative receptor-like protein kinase 2 [Glycine ... | 188 | 3e-46 |
| MA4_82I11.38 | [0] | 66531 | 66985 | hypothetical protein | - | - | - |
| MA4_82I11.40 | [0] | 73795 | 74505 | hypothetical protein | emb|CAB89822.1| tetD [Pseudomonas chlororaphis] | 40 | 0.077 |
| MA4_82I11.42 | [0] | 74983 | 84298 | leucine-rich repeat-containing protein kinase family protein | ref|NP_175749.1| leucine-rich repeat family protein / protein kinase family protein [Arabidopsis thaliana] | 998 | 0.0 |
| MA4_82I11.44 | [1] | 88211 | 89423 | pseudogene, pectinesterase-related | dbj|BAD94663.1| pectinesterase like protein [Arabidopsis thaliana] | 50 | 5e-04 |
| MA4_82I11.46 | [0] | 91163 | 92588 | gibberellin 20-oxidase family protein | gb|AAN73384.1| putative gibberellin 20 oxidase [Oryza rufipogon] | 430 | e-118 |
| MA4_82I11.48 | [0] | 94720 | 101962 | glucose-inhibited division A family protein | ref|NP_001045465.1| Os01g0960300 [Oryza sativa (japonica cultiva... | 942 | 0.0 |
| MA4_54B05.2 | [0] | 1500 | 3397 | proteasome subunit alpha type 2 (20S proteasome alpha subunit B) (proteasome endopeptidase complex), putative | gb|ABB16986.1| unknown [Solanum tuberosum] | 119 | 4e-26 |
| MA4_54B05.4 | [1] | 6641 | 8962 | transposon-related (Ty1-Copia) | emb|CAN68020.1| hypothetical protein [Vitis vinifera] | 91 | 4e-16 |
| MA4_54B05.6 | [1] | 11504 | 12136 | pseudogene, hypothetical protein | - | - | - |
| MA4_54B05.8 | [1] | 12893 | 15853 | transposon-related (monkey) | gb|AAW28578.1| Putative gag-pol polyprotein, identical [Solanum demissum] | 375 | e-101 |
| MA4_54B05.10 | [0] | 17073 | 19808 | hypothetical protein | - | - | - |
| MA4_54B05.12 | [1] | 22010 | 22735 | transposon-related (Ty3-Gypsy) | emb|CAN77247.1| hypothetical protein [Vitis vinifera] | 207 | 9e-52 |
| MA4_54B05.14 | [1] | 23120 | 24433 | pseudogene, hypothetical protein | - | - | - |
| MA4_54B05.16 | [0] | 24831 | 27538 | spliceosomal U5 snRNP-specific 15 kDa protein, putative | gb|AAP85544.1| putative DIM-like protein [Glycine max] | 211 | 2e-53 |
| MA4_54B05.18 | [0] | 28195 | 28879 | hypothetical protein | ref|XP_001112863.1| PREDICTED: coiled-coil domain containing 9 [Macaca mulatta] | 36 | 0.78 |
| MA4_54B05.20 | [1] | 29723 | 33835 | transposon-related (Ty1-Copia) | emb|CAN71183.1| hypothetical protein [Vitis vinifera] | 1439 | 0.0 |
| MA4_54B05.22 | [0] | 35876 | 36469 | isochorismatase hydrolase family protein | emb|CAE05643.2| OSJNBa0038O10.9 [Oryza sativa (japonica cultivar-group)] | 278 | 1e-73 |
| MA4_54B05.24 | [0] | 38373 | 42558 | serine carboxypeptidase (carboxypeptidase D), putative | ref|NP_001047514.1| Os02g0634700 [Oryza sativa (japonica cultivar-group)] | 753 | 0.0 |
| MA4_54B05.26 | [0] | 43502 | 44609 | hypothetical protein | gb|EAY91063.1| hypothetical protein OsI_012296 [Oryza sativa (indica cultivar-group)] | 42 | 0.050 |
| MA4_54B05.28 | [0] | 46631 | 52083 | hypothetical protein | gb|EAZ23916.1| hypothetical protein OsJ_007399 [Oryza sativa (japonica cultivar-group)] | 489 | e-136 |
| MBP_81C12.2 | [1] | 18 | 3777 | transposon-related (Ty1-Copia) | emb|CAN66863.1| hypothetical protein [Vitis vinifera] | 172 | 2e-40 |
| MBP_81C12.4 | [0] | 3825 | 4573 | hypothetical protein | - | - | - |
| MBP_81C12.6 | [0] | 5185 | 24487 | trehalose-6-phosphate synthase, putative | gb|EAY98715.1| hypothetical protein OsI_019948 [Oryza sativa (indica cultivar-group)] | 1320 | 0.0 |
| MBP_81C12.8 | [0] | 24524 | 25858 | hypothetical protein | - | - | - |
| MBP_81C12.10 | [0] | 27284 | 28386 | hypothetical protein | - | - | - |
| MBP_81C12.12 | [0] | 30486 | 31281 | hypothetical protein | - | - | - |
| MBP_81C12.14 | [0] | 31850 | 34406 | hypothetical protein | ref|NP_001055135.1| Os05g0301600 [Oryza sativa (japonica cultivar-group)] | 189 | 7e-47 |
| MBP_81C12.16 | [0] | 38859 | 40999 | GDSL-motif lipase/hydrolase family protein | gb|EAY75691.1| hypothetical protein OsI_003538 [Oryza sativa (indica cultivar-group)] | 490 | e-137 |
| MBP_81C12.18 | [0] | 43094 | 53359 | protein kinase, putative | dbj|BAD86970.1| putative ankyrin-kinase [Oryza sativa (japonica cultivar-group)] | 565 | e-159 |
| MBP_81C12.20 | [0] | 54144 | 54862 | hypothetical protein | - | - | - |
| MBP_81C12.22 | [0] | 55234 | 60001 | hypothetical protein | ref|NP_001044243.1| Os01g0748900 [Oryza sativa (japonica cultivar-group)] | 666 | 0.0 |
| MBP_81C12.24 | [0] | 62778 | 63733 | hypothetical protein | - | - | - |
| MBP_81C12.26 | [0] | 64320 | 70737 | protein kinase, putative | gb|AAU10526.1| putative receptor-like protein kinase 2 [Glycine max] | 634 | e-180 |
| MBP_81C12.28 | [0] | 73039 | 74792 | hypothetical protein | - | - | - |
| MBP_81C12.30 | [0] | 75712 | 76891 | hypothetical protein | gb|EAT42137.1| methyltransferase, putative [Aedes aegypti] | 37 | 0.53 |
| MBP_81C12.32 | [0] | 77849 | 78935 | hypothetical protein | emb|CAA04644.1| purple acid phosphatase precursor [Phaseolus vulgaris] | 37 | 0.96 |
| MBP_81C12.34 | [0] | 86798 | 87088 | leucine rich repeat family protein | gb|AAF78445.1|AC018748_24 Contains a weak similarity to disease resistance protein (cf-5) gene from Lycopersicon esculentum gb|AF053993 [Arabidopsis thaliana] | 55 | 1e-06 |
| MBP_81C12.36 | [0] | 91003 | 94849 | leucine-rich repeat-containing protein / serine/threonine protein kinase-related | gb|AAU10526.1| putative receptor-like protein kinase 2 [Glycine max] | 216 | 2e-54 |
| MBP_81C12.38 | [1] | 99224 | 99635 | hypothetical protein |  |  |  |
| MBP_81C12.40 | [0] | 100303 | 110141 | protein kinase, putative | ref|NP_175749.1| leucine-rich repeat family protein / protein kinase family protein [Arabidopsis thaliana] | 994 | 0.0 |
| MBP_81C12.42 | [0] | 114085 | 114715 | hypothetical protein | gb|AAZ67549.1| 52O08_4 [Brassica rapa subsp. pekinensis] | 57 | 7e-07 |
| MBP_81C12.44 | [0] | 117010 | 118423 | gibberellin 20-oxidase, putative | gb|AAN73384.1| putative gibberellin 20 oxidase [Oryza rufipogon] | 433 | e-119 |
| MBP_81C12.46 | [0] | 120564 | 130117 | glucose-inhibited division A family protein | ref|NP_001045465.1| Os01g0960300 [Oryza sativa (japonica cultivar-group)] | 1056 | 0.0 |
| MBP_81C12.48 | [0] | 131641 | 133804 | DNA-directed RNA polymerase II 8.2 kDa polypeptide, putative | ref|NP_001066312.1| Os12g0180400 [Oryza sativa (japonica cultivar-group)] | 145 | 5e-34 |
| MBP_81C12.50 | [0] | 134279 | 136120 | transcriptional regulator Sir2 family protein | ref|NP_001066310.1| Os12g0179800 [Oryza sativa (japonica cultivar-group)] | 86 | 3e-15 |
| MBP_81C12.52 | [1] | 137546 | 137746 | pseudogene, photosystem I P700 chlorophyll A apoprotein-related | ref|YP_588117.1| photosystem I P700 apoprotein A1 [Helianthus annuus] | 50 | 5e-05 |
| MBP_81C12.54 | [0] | 138585 | 141981 | transcriptional regulator Sir2 family protein | gb|EAY82467.1| hypothetical protein OsI_036426 [Oryza sativa (indica cultivar-group)] | 259 | 2e-67 |
| MBP_91N22.2 | [0] | 3 | 1670 | hypothetical protein, 3' partial | emb|CAN80751.1| hypothetical protein [Vitis vinifera] | 141 | 2e-32 |
| MBP_91N22.4 | [0] | 5909 | 8250 | hypothetical protein | emb|CAN83082.1| hypothetical protein [Vitis vinifera] | 251 | 3e-65 |
| MBP_91N22.6 | [0] | 8663 | 12575 | ubiquitin ligase SINAT5-related (seven in absentia protein family) | emb|CAN75137.1| hypothetical protein [Vitis vinifera] | 549 | e-154 |
| MBP_91N22.8 | [0] | 14064 | 15893 | mitochondrial ATP synthase g subunit family protein | ref|NP_001043488.1| Os01g0600000 [Oryza sativa (japonica cultivar-group)] | 216 | 2e-55 |
| MBP_91N22.10 | [0] | 16832 | 19744 | hypothetical protein | gb|EAY74823.1| hypothetical protein OsI_002670 [Oryza sativa (indica cultivar-group)] | 280 | 2e-73 |
| MBP_91N22.12 | [0] | 20442 | 21245 | chlorophyll A-B binding protein (CAB), putative | gb|ABF17940.1| putative chloroplast chlorophyll a/b-binding protein [Carya cathayensis] | 496 | e-139 |
| MBP_91N22.14 | [0] | 22303 | 23220 | serine O-acetyltransferase, putative | gb|AAF19000.1|AF212156_1 serine acetyltransferase [Allium cepa] | 429 | e-118 |
| MBP_91N22.16 | [1] | 23872 | 27743 | transposon-related (Ty3-Gypsy) | emb|CAN68662.1| hypothetical protein [Vitis vinifera] | 127 | 1e-26 |
| MBP_91N22.18 | [0] | 28603 | 32875 | protein kinase, putative | gb|EAY98752.1| hypothetical protein OsI_019985 [Oryza sativa (indica cultivar-group)] | 892 | 0.0 |
| MBP_91N22.20 | [0] | 33138 | 33646 | hypothetical protein | ref|XP_001630177.1| predicted protein [Nematostella vectensis] | 36 | 0.78 |
| MBP_91N22.22 | [0] | 34063 | 37740 | dual specificity protein phosphatase family protein | emb|CAN61438.1| hypothetical protein [Vitis vinifera] | 328 | 1e-87 |
| MBP_91N22.24 | [0] | 38335 | 40437 | hypothetical protein | ref|ZP_01110499.1| polar flagellar hook-length control protein FliK [Alteromonas macleodii 'Deep ecotype'] | 46 | 0.008 |
| MBP_91N22.26 | [0] | 40502 | 42302 | protein kinase family protein | gb|EAY75768.1| hypothetical protein OsI_003615 [Oryza sativa (indica cultivar-group)] | 88 | 1e-16 |
| MBP_91N22.28 | [0] | 43887 | 48685 | protein kinase family protein | gb|EAZ13487.1| hypothetical protein OsJ_003312 [Oryza sativa (japonica cultivar-group)] | 513 | e-143 |
| MBP_91N22.30 | [1] | 49055 | 50809 | pseudogene, hypothetical protein | - | - | - |
| MBP_91N22.32 | [0] | 51563 | 56210 | protein phosphatase 2C, putative | emb|CAN71518.1| hypothetical protein [Vitis vinifera] | 377 | e-103 |
| MBP_91N22.34 | [0] | 56517 | 57642 | hypothetical protein | ref|XP_001210095.1| conserved hypothetical protein [Aspergillus terreus NIH2624] | 37 | 0.29 |
| MBP_91N22.36 | [0] | 57806 | 59366 | transcriptional factor B3 family protein / AT hook motif-containing protein | dbj|BAB89497.1| hypothetical protein [Oryza sativa (japonica cultivar-group)] | 216 | 2e-54 |
| MBP_91N22.38 | [0] | 60080 | 60856 | DNA-binding protein-related | ref|NP_001049178.1| Os03g0183100 [Oryza sativa (japonica cultivar-group)] | 150 | 1e-34 |
| MBP_91N22.40 | [0] | 63248 | 65678 | dioxygenase-related protein | gb|AAW59435.1| decreased apical dominance protein [Petunia [Petunia x hybrida] | 845 | 0.0 |
| MBP_91N22.42 | [0] | 65873 | 67099 | hypothetical protein | - | - | - |
| MBP_91N22.44 | [0] | 68425 | 69372 | AP2/EREBP transcription factor, putative | ref|NP_196720.1| TINY2 (TINY2); DNA binding / transcription factor [Arabidopsis thaliana] | 147 | 2e-33 |
| MBP_91N22.46 | [0] | 70558 | 72461 | hypothetical protein | - | - | - |
| MBP_91N22.48 | [1] | 77197 | 77937 | transposon-related (Ty1-Copia) | emb|CAN72461.1| hypothetical protein [Vitis vinifera] | 155 | 2e-36 |
| MBP_91N22.50 | [1] | 80184 | 80481 | hypothetical protein | - | - | - |
| MBP_91N22.52 | [0] | 80495 | 81956 | glycosyl transferase family 14 protein | ref|NP_194478.3| glycosyltransferase family 14 protein / core-2/I-branching enzyme family protein [Arabidopsis thaliana] | 567 | e-160 |
| MBP_91N22.54 | [0] | 82909 | 86138 | dynamin family protein | dbj|BAD86966.1| dynamin-like [Oryza sativa (japonica cultivar-group)] | 1135 | 0.0 |
| MBP_91N22.56 | [0] | 86486 | 86851 | hypothetical protein | ref|NP_001056059.1| Os05g0518800 [Oryza sativa (japonica cultivar-group)] | 79 | 1e-13 |
| MBP_91N22.58 | [0] | 87167 | 87995 | hypothetical protein | - | - | - |
| MBP_91N22.60 | [0] | 90197 | 96753 | protein kinase family protein / ankyrin repeat family protein | dbj|BAD86970.1| putative ankyrin-kinase [Oryza sativa (japonica cultivar-group)] | 561 | e-158 |
| MBP_91N22.62 | [0] | 99875 | 100532 | GTP-binding protein, putative | gb|AAT64023.1| putative GTP-binding protein [Gossypium hirsutum] | 144 | 1e-33 |
| MBP_91N22.64 | [1] | 101319 | 105284 | transposon-related (Ty1-Copia) | gb|AAT85194.1| putative polyprotein [Oryza sativa (japonica cultivar-group)] | 1268 | 0.0 |
| MBP_91N22.66 | [0] | 107371 | 108196 | GTP-binding protein, putative | emb|CAN75016.1| hypothetical protein [Vitis vinifera] | 230 | 6e-59 |
| MBP_91N22.68 | [0] | 109364 | 110977 | leucine rich repeat family protein | gb|AAT64029.1| putative leucine-rich repeat family protein [Gossypium hirsutum] | 324 | 1e-86 |
| MBP_91N22.70 | [0] | 111779 | 114040 | translocase of outer membrane 7 (TOM7) family protein | ref|NP_001043627.1| Os01g0626300 [Oryza sativa (japonica cultivar-group)] | 57 | 7e-06 |
| MBP_91N22.72 | [0] | 116535 | 125887 | transcriptional repressor protein-related | gb|EAZ10246.1| hypothetical protein OsJ_000071 [Oryza sativa (japonica cultivar-group)] | 1484 | 0.0 |
| MBP_91N22.74 | [0] | 126762 | 129534 | hypothetical protein | gb|EAZ01032.1| hypothetical protein OsI_022264 [Oryza sativa (indica cultivar-group)] | 90 | 9e-17 |
| MBP_91N22.76 | [1] | 132078 | 132863 | transposon-related (Ty1-Copia) | - | - | - |
| MBP_91N22.78 | [0] | 134102 | 136554 | protein kinase family protein | ref|NP_001041798.1| Os01g0110500 [Oryza sativa (japonica (japonica cultivar-group)] | 546 | e-153 |
| MBP_91N22.80 | [0] | 137613 | 139868 | exostosin family protein | gb|EAZ10234.1| hypothetical protein OsJ_000059 [Oryza sativa (japonica cultivar-group)] | 559 | e-157 |
| MBP_91N22.82 | [0] | 140349 | 141624 | hypothetical protein | gb|AAL87151.1|AF480496_5 unknown [Oryza sativa (japonica cultivar-group)] | 194 | 8e-48 |
| MBP_91N22.84 | [0] | 144273 | 146490 | glycoside hydrolase family 17 protein | ref|NP_001047883.1| Os02g0708400 [Oryza sativa (japonica cultivar-group)] | 885 | 0.0 |
| MBP_91N22.86 | [0] | 148297 | 149436 | glycoside hydrolase family 17 protein | ref|NP_001063767.1| Os09g0533200 [Oryza sativa (japonica cultivar-group)] | 176 | 3e-42 |
| MBP_91N22.88 | [1] | 153347 | 154213 | hypothetical protein | gb|EAZ24333.1| hypothetical protein OsJ_007816 [Oryza sativa (japonica cultivar-group)] | 68 | 1e-10 |
| MA4_112I10.2 | [1] | 892 | 2712 | hypothetical protein | - | - | - |
| MA4_112I10.4 | [1] | 4152 | 8798 | transposon-related (Ty1-Copia) | emb|CAN76317.1| hypothetical protein [Vitis vinifera] | 1311 | 0.0 |
| MA4_112I10.6 | [0] | 9763 | 12205 | hypothetical protein | ref|XP_001236007.1| PREDICTED: hypothetical protein [Gallus gallus] | 43 | 0.020 |
| MA4_112I10.8 | [1] | 13137 | 13724 | transposon-related (Ty1-Copia) | - | - | - |
| MA4_112I10.10 | [0] | 13897 | 15106 | cysteine protease, putative | gb|AAR92154.1| putative cysteine protease 1 [Iris hollandica] | 417 | e-114 |
| MA4_112I10.12 | [1] | 16651 | 19278 | transposon-related (Ty1-Copia) | - | - | - |
| MA4_112I10.14 | [0] | 19892 | 20634 | hypothetical protein | gb|EAY75918.1| hypothetical protein OsI_003765 [Oryza sativa (indica cultivar-group)] | 49 | 3e-04 |
| MA4_112I10.16 | [0] | 21097 | 24664 | phytochrome, putative | gb|AAP06790.1| phytochrome C1 apoprotein; PhyC1 [Zea mays] | 576 | e-162 |
| MA4_112I10.18 | [0] | 24760 | 25489 | hypothetical protein | - | - | - |
| MA4_112I10.20 | [0] | 32606 | 34430 | N-acetylglucosaminyltransferase, putative | dbj|BAD36053.1| putative beta-1,2-N-acetylglucosaminyltransferase II [Oryza sativa (japonica cultivar-group)] | 605 | e-171 |
| MA4_112I10.22 | [0] | 34790 | 36779 | hypothetical protein | dbj|BAD31146.1| unknown protein [Oryza sativa (japonica cultivar-group)] | 62 | 1e-07 |
| MA4_112I10.24 | [0] | 39759 | 40249 | hypothetical protein | ref|NP_001050596.1| Os03g0595300 [Oryza sativa (japonica cultivar-group)] | 138 | 1e-31 |
| MA4_112I10.26 | [0] | 42044 | 45872 | oligopeptide transporter OPT family protein | gb|AAQ91200.1| putative glutathione transporter [Zea mays] | 1278 | 0.0 |
| MA4_112I10.28 | [0] | 46085 | 46887 | hypothetical protein | - | - | - |
| MA4_112I10.30 | [0] | 46992 | 48445 | hypothetical protein | - | - | - |
| MA4_112I10.32 | [1] | 48813 | 49296 | hypothetical protein | - | - | - |
| MA4_112I10.34 | [0] | 52989 | 54007 | hypothetical protein | ref|XP_364632.2| hypothetical protein MGG_09477 [Magnaporthe grisea 70-15] | 39 | 0.46 |
| MA4_112I10.36 | [1] | 54782 | 55006 | hypothetical protein | - | - | - |
| MA4_112I10.38 | [0] | 55158 | 59405 | pumilio/Puf RNA-binding domain-containing protein | ref|NP_200462.2| APUM12 (ARABIDOPSIS PUMILIO 12); RNA binding [Arabidopsis thaliana] | 115 | 2e-23 |
| MA4_112I10.40 | [0] | 60366 | 61254 | chlorophyll A-B binding protein (CAB), putative | emb|CAN71610.1| hypothetical protein [Vitis vinifera] | 510 | e-143 |
| MA4_112I10.42 | [0] | 61659 | 63344 | hypothetical protein | gb|EAU86197.1| hypothetical protein CC1G_03408 [Coprinopsis cinerea okayama7#130] | 38 | 0.92 |
| MA4_112I10.44 | [0] | 63376 | 63711 | hypothetical protein | emb|CAN83217.1| hypothetical protein [Vitis vinifera] | 89 | 8e-17 |
| MA4_112I10.46 | [0] | 63718 | 65545 | hypothetical protein | - | - | - |
| MA4_112I10.48 | [0] | 65609 | 66818 | hypothetical protein | ref|XP_001496580.1| PREDICTED: similar to alpha-1 type IV collagen [Equus caballus] | 37 | 0.52 |
| MA4_112I10.50 | [0] | 67061 | 69180 | RNA-binding protein, putative | emb|CAN75218.1| hypothetical protein [Vitis vinifera] | 500 | e-139 |
| MA4_112I10.52 | [0] | 70437 | 72648 | hypothetical protein | gb|EAZ27638.1| hypothetical protein OsJ_011121 [Oryza sativa (japonica cultivar-group)] | 292 | 1e-77 |
| MA4_112I10.54 | [0] | 74087 | 74980 | remorin-related | ref|NP_001060036.1| Os07g0569100 [Oryza sativa (japonica cultivar-group)] | 257 | 9e-67 |
| MA4_112I10.56 | [0] | 77402 | 81003 | basic leucine zipper (bZIP) transcription factor family protein | emb|CAB62402.1| maize Em binding protein-1a [Zea mays] | 157 | 7e-37 |
| MA4_112I10.58 | [0] | 81387 | 83218 | hypothetical protein | gb|AAW34261.1| DANA2 [Zea mays] | 42 | 0.025 |
| MA4_112I10.60 | [0] | 86541 | 87286 | hypothetical protein | ref|NP_175131.3| unknown protein [Arabidopsis thaliana] | 235 | 2e-60 |
| MA4_112I10.62 | [0] | 88143 | 90940 | phosphoglycerate kinase, chloroplast, putative | sp|Q42961|PGKH_TOBAC Phosphoglycerate kinase, chloroplast precursor [Nicotiana tabacum] | 699 | 0.0 |
| MA4_112I10.64 | [0] | 94036 | 97610 | hypothetical protein | gb|AAD30220.1|AC007202_2 Is a member of PF|00004 ATPases associated with various cellular activities (AAA) family [Arabidopsis thaliana] | 340 | 1e-91 |
| MA4_112I10.66 | [0] | 99227 | 101298 | FtsH protein, putative | gb|AAD30220.1|AC007202_2 Is a member of PF|00004 ATPases associated with various cellular activities (AAA) family [Arabidopsis thaliana] | 387 | e-106 |
| F71C19.2 | [1] | 739 | 1221 | hypothetical protein | - | - | - |
| F71C19.4 | [0] | 4062 | 10471 | ribophorin I family protein | gb|EAY96183.1| hypothetical protein OsI_017416 [Oryza sativa (indica cultivar-group)] | 622 | e-176 |
| F71C19.6 | [0] | 11995 | 14863 | glycosyl transferase family 2 protein | emb|CAN61137.1| hypothetical protein [Vitis vinifera] | 1386 | 0.0 |
| F71C19.8 | [0] | 17264 | 19354 | epsin N-terminal homology (ENTH) domain-containing protein | dbj|BAD19388.1| epsin-like [Oryza sativa (japonica cultivar-group)] | 147 | 2e-33 |
| F71C19.10 | [0] | 21586 | 24154 | epsin N-terminal homology (ENTH) domain-containing protein | gb|AAK98696.1|AC069158_8 Putative epsin [Oryza sativa] | 305 | 8e-81 |
| F71C19.12 | [0] | 29386 | 31885 | hypothetical protein | ref|NP_181258.2| unknown protein [Arabidopsis thaliana] | 327 | 5e-88 |
| F71C19.14 | [0] | 34768 | 37419 | pentatricopeptide (PPR) repeat containing protein, putative | gb|EAY84160.1| hypothetical protein OsI_005393 [Oryza sativa (indica cultivar-group)] | 898 | 0.0 |
| F71C19.16 | [1] | 47992 | 52087 | transposon-related (monkey) | emb|CAN69702.1| hypothetical protein [Vitis vinifera] | 219 | 9e-55 |
| F71C19.18 | [1] | 55099 | 55341 | hypothetical protein | - | - | - |
| F71C19.20 | [0] | 55781 | 58032 | phenylalanine ammonia-lyase, putative | gb|ABM67591.1| phenylalanine ammonia-lyase [Vitis vinifera] | 1124 | 0.0 |
| F71C19.22 | [0] | 58333 | 59186 | TCP family transcription factor | emb|CAE45599.1| TCP-domain protein [Antirrhinum majus subsp. majus] | 43 | 0.026 |
| F71C19.24 | [0] | 60143 | 67561 | hypothetical protein | dbj|BAD67948.1| unknown protein [Oryza sativa (japonica cultivar-group)] | 101 | 6e-19 |
| F71C19.26 | [0] | 75953 | 78692 | transcriptional regulator (MOM1-related) | gb|EAZ21463.1| hypothetical protein OsJ_004946 [Oryza sativa (japonica cultivar-group)] | 119 | 3e-25 |
| F71C19.28 | [1] | 80419 | 85015 | transposon-related (Banana streak virus) | ref|YP_233107.1| polyprotein [Banana streak GF virus] | 1846 | 0.0 |
| F71C19.30 | [1] | 86255 | 89101 | transposon-related (Banana streak virus) | ref|YP_233107.1| polyprotein [Banana streak GF virus] | 1251 | 0.0 |
| F71C19.32 | [1] | 90068 | 93854 | transposon-related (Banana streak virus) | ref|YP_233107.1| polyprotein [Banana streak GF virus] | 1836 | 0.0 |
| F71C19.34 | [1] | 93997 | 95418 | transposon-related (Ty3-Gypsy) | emb|CAN68669.1| hypothetical protein [Vitis vinifera] | 278 | 9e-73 |
| F71C19.36 | [0] | 98896 | 101289 | transcriptional regulator (MOM1-related) | gb|EAY84163.1| hypothetical protein OsI_005396 [Oryza sativa (indica cultivar-group)] | 259 | 2e-67 |
| F71C19.38 | [1] | 102180 | 104653 | pseudogene, hypothetical protein | ref|NP_680299.1| hAT dimerisation domain-containing protein [Arabidopsis thaliana] | 123 | 4e-26 |
| F71C19.40 | [0] | 107736 | 111174 | hypothetical protein | gb|EAZ21463.1| hypothetical protein OsJ_004946 [Oryza sativa (japonica cultivar-group)] | 127 | 1e-27 |
| F71C19.42 | [0] | 112684 | 117441 | hypothetical protein | emb|CAN64222.1| hypothetical protein [Vitis vinifera] | 60 | 7e-07 |
| F71C19.44 | [1] | 118473 | 118806 | hypothetical protein | - | - | - |
| F71C19.46 | [0] | 122614 | 123216 | auxin-responsive SAUR family protein | emb|CAE05452.3| OSJNBa0073E02.12 [Oryza sativa (japonica cultivar-group)] | 80 | 9e-14 |
| F71C19.48 | [0] | 125042 | 127808 | actin-depolymerizing factor, putative | gb|AAF60173.1|AF236068_1 actin depolymerizing factor [Elaeis guineensis] | 224 | 2e-57 |
| F71C19.50 | [1] | 128615 | 129077 | hypothetical protein | - | - | - |
