# Supplementary material for: Insights into the Musa genome: Syntenic relationships to rice and between Musa species
Source: BMC Genomics. 2008 Jan 30;9:58. doi: 10.1186/1471-2164-9-58 (PMC2270835; doi:10.1186/1471-2164-9-58)
Supplement: Additional file 5 — Supplementary Table 5. List of genes involved in synteny relationship between Musa and rice based on i-ADHoRE results. Multiplicon is a BAC genomic sequence on which the baseclusters are isolated and represents a cluster of 3 genes minima. [file 1471-2164-9-58-S5.doc]

**Supplementary Table 5.**

| **Multiplicon** | **Basecluster** | ***Musa* genes** | **Rice genes** |
| --- | --- | --- | --- |
| 1 | 1 | MA4_8L21.38 | LOC_Os03g55780 |
| 1 | 1 | MA4_8L21.42 | LOC_Os03g55790 |
| 1 | 1 | MA4_8L21.44 | LOC_Os03g55820 |
| 1 | 1 | MA4_8L21.48 | LOC_Os03g55850 |
| 1 | 2 | MA4_8L21.6 | LOC_Os03g55330 |
| 1 | 2 | MA4_8L21.8 | LOC_Os03g55350 |
| 1 | 2 | MA4_8L21.20 | LOC_Os03g55450 |
| 1 | 2 | MA4_8L21.22 | LOC_Os03g55470 |
| 1 | 2 | MA4_8L21.26 | LOC_Os03g55550 |
| 1 | 2 | MA4_8L21.36 | LOC_Os03g55610 |
| 2 | 3 | MA4_42M13.2 | LOC_Os02g07220 |
| 2 | 3 | MA4_42M13.6 | LOC_Os02g07230 |
| 2 | 3 | MA4_42M13.10 | LOC_Os02g07240 |
| 3 | 4 | MA4_54B05.22 | At5g23220 |
| 3 | 4 | MA4_54B05.24 | At5g23210 |
| 3 | 4 | MA4_54B05.28 | At5g23200 |
| 4 | 5 | MA4_112I10.10 | LOC_Os03g54130 |
| 4 | 5 | MA4_112I10.16 | LOC_Os03g54080 |
| 4 | 5 | MA4_112I10.22 | LOC_Os03g54050 |
| 5 | 6 | MA4_25J11.4 | LOC_Os05g49230 |
| 5 | 6 | MA4_25J11.8 | LOC_Os05g49220 |
| 5 | 6 | MA4_25J11.50 | LOC_Os05g49070 |
| 5 | 6 | MA4_25J11.52 | LOC_Os05g49050 |
| 6 | 7 | MBP_91N22.8 | LOC_Os01g52200 |
| 6 | 7 | MBP_91N22.12 | LOC_Os01g52240 |
| 6 | 7 | MBP_91N22.14 | LOC_Os01g52260 |
| 6 | 8 | MBP_91N22.40 | LOC_Os01g54270 |
| 6 | 8 | MBP_91N22.54 | LOC_Os01g54420 |
| 6 | 8 | MBP_91N22.60 | LOC_Os01g54480 |
| 6 | 8 | MBP_91N22.68 | LOC_Os01g54630 |
| 6 | 9 | MBP_91N22.72 | LOC_Os01g01960 |
| 6 | 9 | MBP_91N22.74 | LOC_Os01g01970 |
| 6 | 9 | MBP_91N22.78 | LOC_Os01g02040 |
| 7 | 10 | MA4_106O17.2 | LOC_Os04g53310 |
| 7 | 10 | MA4_106O17.6 | LOC_Os04g53230 |
| 7 | 10 | MA4_106O17.10 | LOC_Os04g53190 |
| 8 | 11 | MuH9-3 | LOC_Os04g41340 |
| 8 | 11 | MuH9-5 | LOC_Os04g41470 |
| 8 | 11 | MuH9-7 | LOC_Os04g41540 |
| 9 | 12 | MuG9-3 | LOC_Os04g59310 |
| 9 | 12 | MuG9-5 | LOC_Os04g59320 |
| 9 | 12 | MuG9-7 | LOC_Os04g59340 |
