# Supplementary material for: Insights into the Musa genome: Syntenic relationships to rice and between Musa species
Source: BMC Genomics. 2008 Jan 30;9:58. doi: 10.1186/1471-2164-9-58 (PMC2270835; doi:10.1186/1471-2164-9-58)
Supplement: Additional file 6 — Supplementary Figure 1. Musa-rice syntenic region between MuH9 BAC clone and rice chromosome 4. Homologous genes between Musa and rice are indicated by shaded areas. Genes annotated such as hypothetical genes are white. [file 1471-2164-9-58-S6.ppt]

## Slide 1
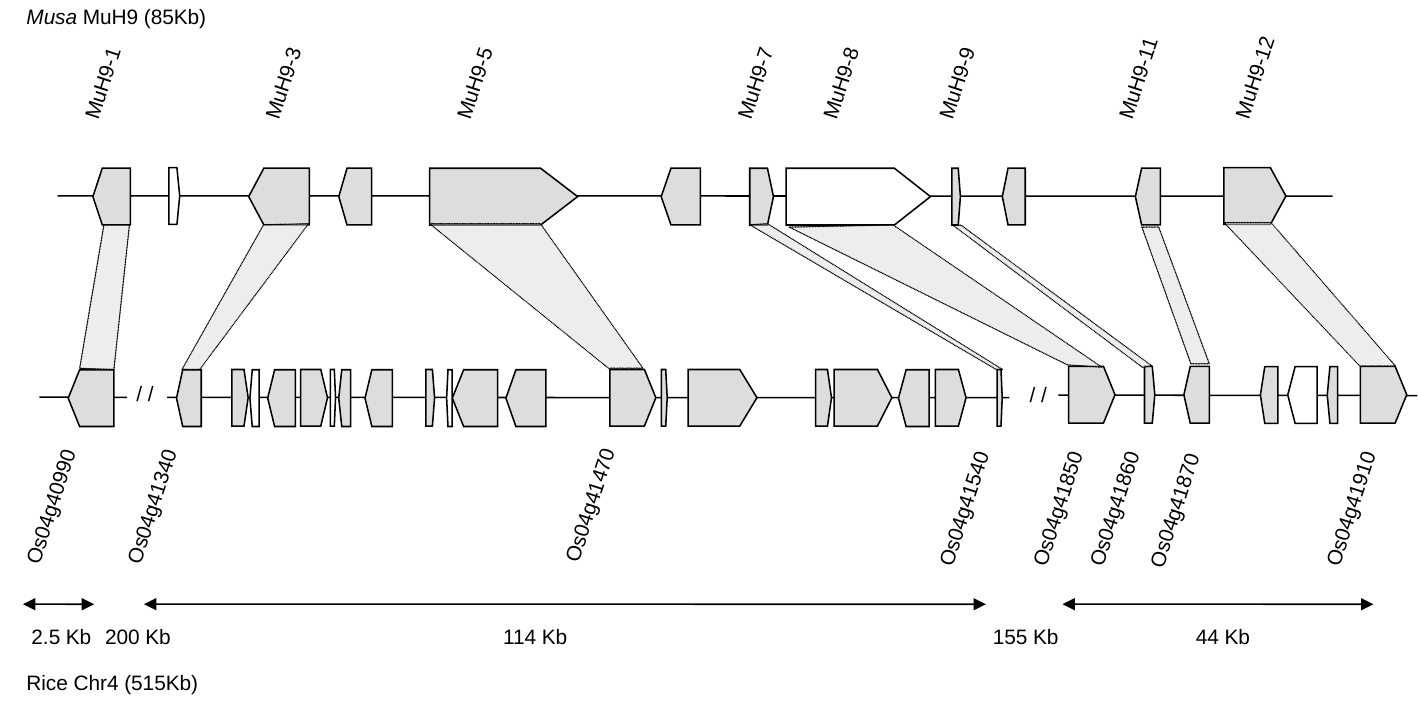

MuH9-12
MuH9-9
MuH9-3
MuH9-1
MuH9-5
MuH9-11
MuH9-8
MuH9-7
Musa MuH9 (85Kb)
/ /
/ /
Os04g41470
Os04g41340
Os04g40990
Os04g41910
Os04g41540
Os04g41850
Os04g41860
Os04g41870
2.5 Kb
200 Kb
114 Kb
155 Kb
44 Kb
Rice Chr4 (515Kb)
