# Supplementary material for: Insights into the Musa genome: Syntenic relationships to rice and between Musa species
Source: BMC Genomics. 2008 Jan 30;9:58. doi: 10.1186/1471-2164-9-58 (PMC2270835; doi:10.1186/1471-2164-9-58)
Supplement: Additional file 7 — Supplementary Figure 2. The Musa-rice syntenic region around the highly conserved porphobilinogen deaminase gene. Shaded areas connect homologous genes conserved between chromosome 2 of rice and Musa MA4_42M13 BAC clone (isolated by SbRPG748 probe). Genes annotated such as hypothetical genes are white. In Musa, a recent local duplication of the porphobilinogen deaminase gene occurred (genes MA4_42M13.6 and MA4_42M13.8). [file 1471-2164-9-58-S7.ppt]

## Slide 1
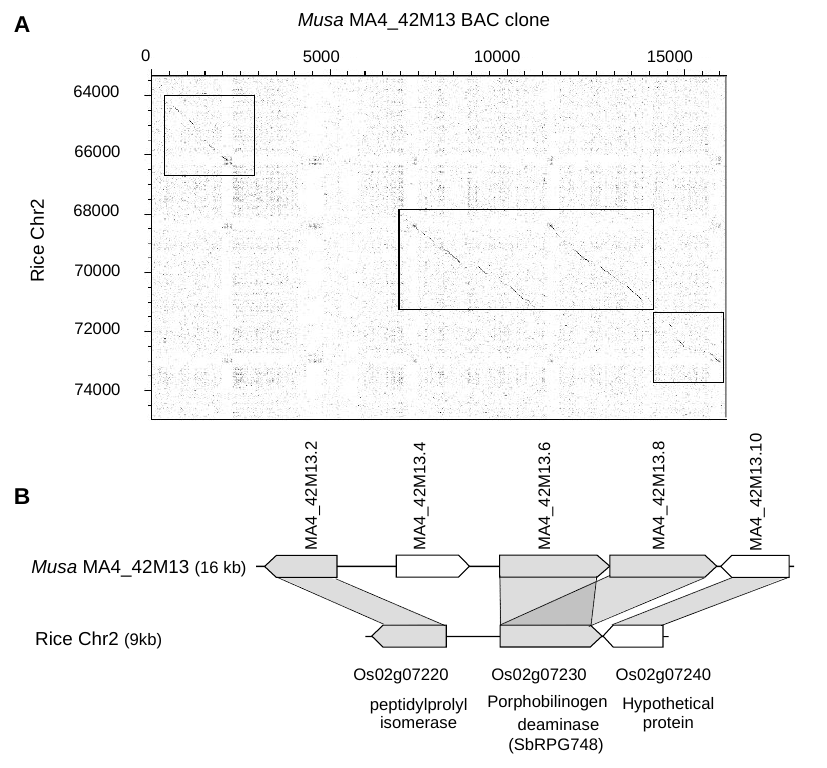

Musa MA4_42M13 BAC clone
A
0
10000
15000
5000
64000
66000
68000
Rice Chr2
70000
72000
74000
MA4_42M13.10
B
MA4_42M13.2
MA4_42M13.8
MA4_42M13.4
MA4_42M13.6
Musa MA4_42M13 (16 kb)
Rice Chr2 (9kb)
Os02g07220
peptidylprolyl
isomerase
Os02g07230
Porphobilinogen
 deaminase
(SbRPG748)
Os02g07240
Hypothetical
protein
