# Supplementary material for: Insights into the Musa genome: Syntenic relationships to rice and between Musa species
Source: BMC Genomics. 2008 Jan 30;9:58. doi: 10.1186/1471-2164-9-58 (PMC2270835; doi:10.1186/1471-2164-9-58)
Supplement: Additional file 8 — Supplementary Figure 3. Phylogenetic analyses on the seven of the ten M. acuminata genes from MA4_25J11 BAC clone. These seven Musa genes have homologous genes in rice chromosomes 1 and 5 and the locus numbers are available on Figure 5B. MA4_25J11 BAC clone was isolated by SbRPG132 probe. [file 1471-2164-9-58-S8.ppt]

## Slide 1
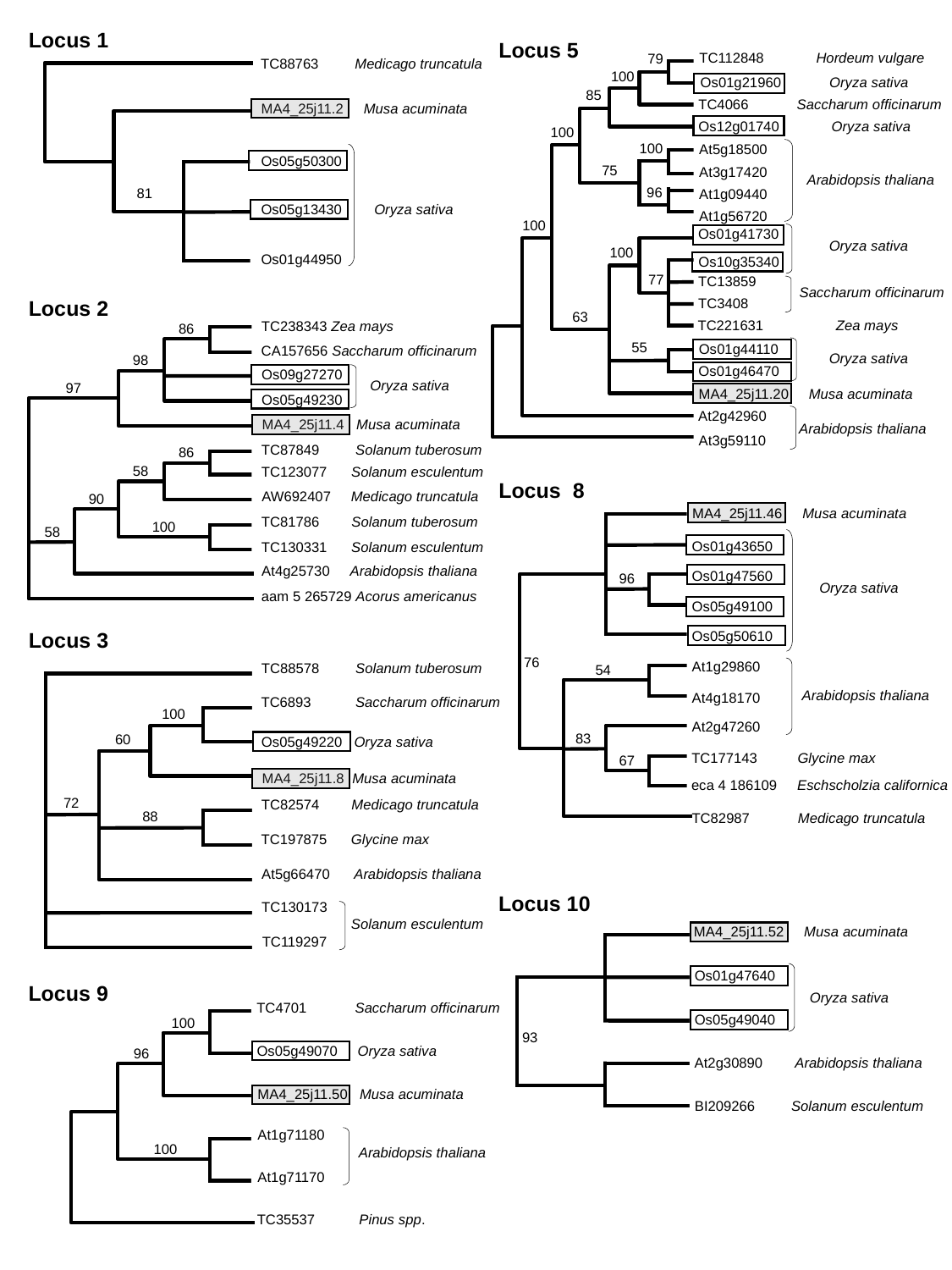

Locus 1
Locus 5
TC112848 Hordeum vulgare
79
TC88763 Medicago truncatula
100
Os01g21960 Oryza sativa
85
TC4066 Saccharum officinarum
MA4_25j11.2 Musa acuminata
Os12g01740 Oryza sativa
100
100
At5g18500
Os05g50300
75
At3g17420
 Arabidopsis thaliana
96
81
At1g09440
Oryza sativa
Os05g13430
At1g56720
100
Os01g41730
Oryza sativa
100
Os01g44950
Os10g35340
77
TC13859
 Saccharum officinarum
Locus 2
TC3408
63
TC221631 Zea mays
TC238343 Zea mays
86
55
Os01g44110
CA157656 Saccharum officinarum
Oryza sativa
98
Os01g46470
Os09g27270
Oryza sativa
97
MA4_25j11.20 Musa acuminata
Os05g49230
At2g42960
 Arabidopsis thaliana
MA4_25j11.4 Musa acuminata
At3g59110
TC87849 Solanum tuberosum
86
58
TC123077 Solanum esculentum
Locus 8
AW692407 Medicago truncatula
90
MA4_25j11.46 Musa acuminata
TC81786 Solanum tuberosum
100
58
Os01g43650
TC130331 Solanum esculentum
At4g25730 Arabidopsis thaliana
Os01g47560
96
Oryza sativa
aam 5 265729 Acorus americanus
Os05g49100
Locus 3
Os05g50610
76
At1g29860
TC88578 Solanum tuberosum
54
 Arabidopsis thaliana
At4g18170
TC6893 Saccharum officinarum
100
At2g47260
83
60
Os05g49220 Oryza sativa
TC177143 Glycine max
67
MA4_25j11.8 Musa acuminata
eca 4 186109 Eschscholzia californica
72
TC82574 Medicago truncatula
88
TC82987 Medicago truncatula
TC197875 Glycine max
At5g66470 Arabidopsis thaliana
Locus 10
TC130173
 Solanum esculentum
MA4_25j11.52 Musa acuminata
TC119297
Os01g47640
Locus 9
Oryza sativa
TC4701 Saccharum officinarum
Os05g49040
100
93
Os05g49070 Oryza sativa
96
At2g30890 Arabidopsis thaliana
MA4_25j11.50 Musa acuminata
BI209266 Solanum esculentum
At1g71180
 Arabidopsis thaliana
100
At1g71170
TC35537 Pinus spp.
