# Supplementary figures and images for: Insights into the Musa genome: Syntenic relationships to rice and between Musa species
Source: BMC Genomics. 2008 Jan 30;9:58. doi: 10.1186/1471-2164-9-58 (PMC2270835; doi:10.1186/1471-2164-9-58)

## Slide 1
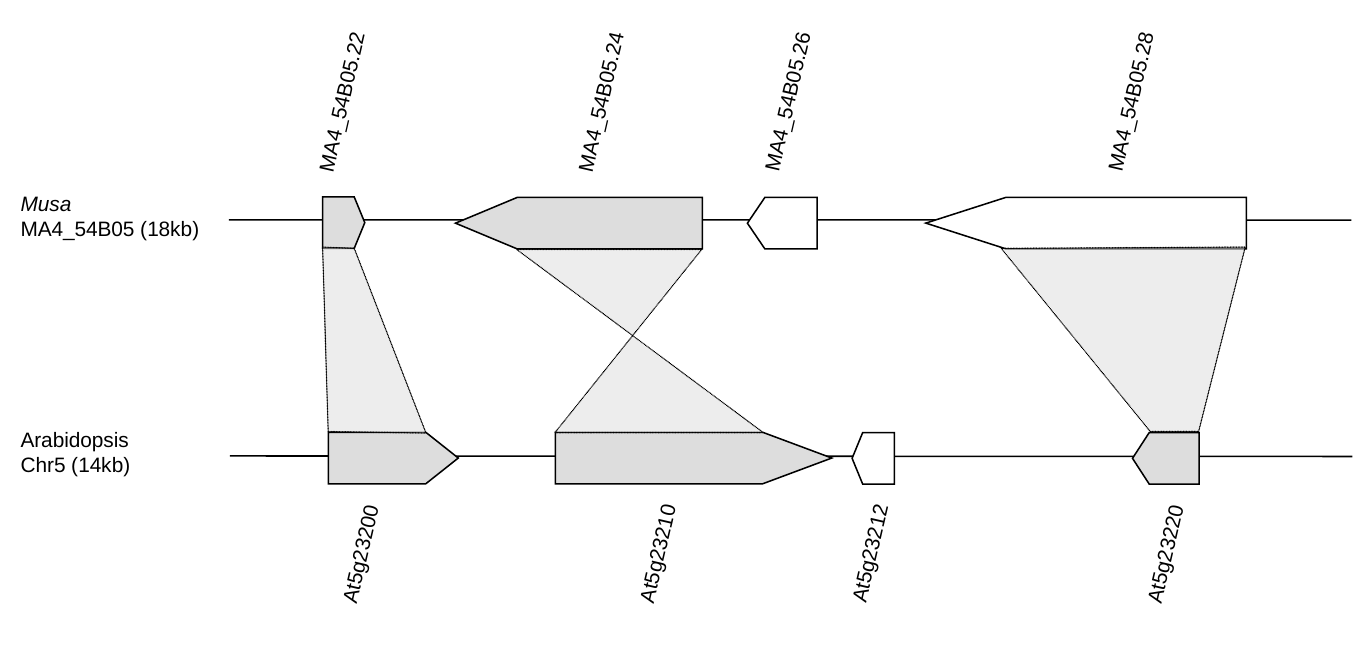

MA4_54B05.22
MA4_54B05.26
MA4_54B05.24
MA4_54B05.28
Musa
MA4_54B05 (18kb)
Arabidopsis
Chr5 (14kb)
At5g23212
At5g23210
At5g23200
At5g23220

Supplement: Additional file 9 — Supplementary Figure 4. Musa-Arabidopsis syntenic region between Musa MA4_54B05 BAC clone and Arabidopsis chromosome 5. Homologous genes between Musa and Arabidopsis are indicated by shaded areas. Genes annotated such as hypothetical genes are white. MA4_54B05 BAC clone was isolated by SbRPG661 probe. [file 1471-2164-9-58-S9.ppt]
