# Supplementary material for: Insights into the Musa genome: Syntenic relationships to rice and between Musa species
Source: BMC Genomics. 2008 Jan 30;9:58. doi: 10.1186/1471-2164-9-58 (PMC2270835; doi:10.1186/1471-2164-9-58)
Supplement: Additional file 10 — Supplementary Figure 5. Collinearity between M. acuminata (MA4_54N07) and M. balbisiana (MBP_91N22) around the CIR560 marker. The shaded areas connecting the two genomic regions represent conserved genes. Predicted genes and their orientation in each Musa BAC clone are shown as boxed areas. The genes for which the name is in bold hybridize with the marker. Genes annotated such as hypothetical genes are white. (A) Dot plot analysis of the two pairs of homeologous BACs from M. acuminata and M. balbisiana. (B) Diagram of the syntenic regions between the two BAC clones. [file 1471-2164-9-58-S10.ppt]

## Slide 1
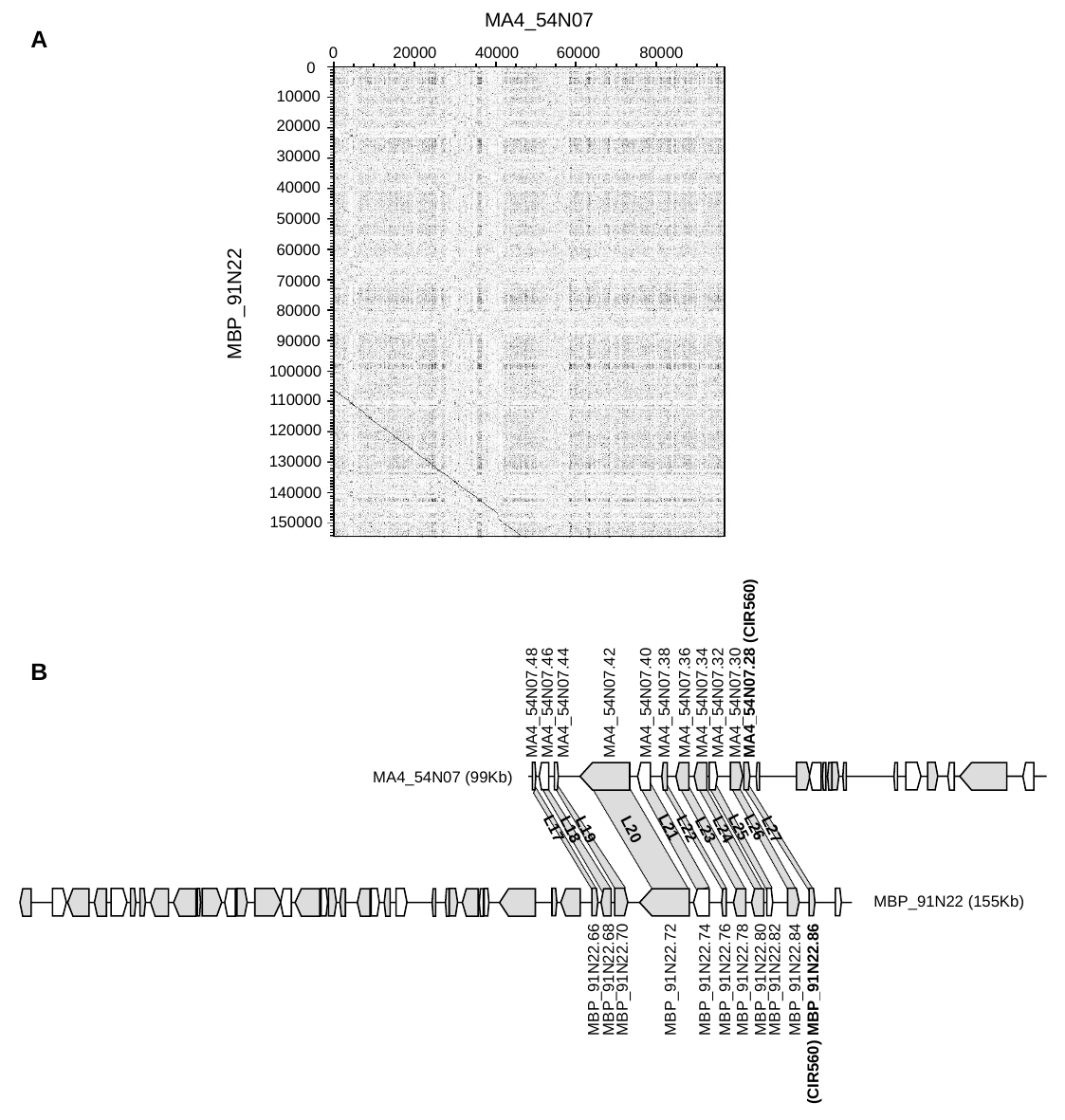

MA4_54N07
A
0
20000
40000
60000
80000
0
10000
20000
30000
40000
50000
60000
70000
MBP_91N22
80000
90000
100000
110000
120000
130000
140000
150000
MA4_54N07.48
MA4_54N07.46
MA4_54N07.44
MA4_54N07.42
MA4_54N07.40
MA4_54N07.38
MA4_54N07.36
MA4_54N07.34
MA4_54N07.32
MA4_54N07.30
MA4_54N07.28 (CIR560)
B
MA4_54N07 (99Kb)
L21
L25
L26
L17
L22
L18
L19
L20
L24
L27
L23
MBP_91N22 (155Kb)
MBP_91N22.66
MBP_91N22.68
MBP_91N22.70
MBP_91N22.72
MBP_91N22.74
MBP_91N22.76
MBP_91N22.78
MBP_91N22.80
MBP_91N22.82
MBP_91N22.84
(CIR560) MBP_91N22.86
